# Baicalin improves the inflammatory response of RA-FLS by targeting the circ_0000734/miR-197-5p/IKBKB axis

**DOI:** 10.3389/fimmu.2026.1828868

**Published:** 2026-05-28

**Authors:** Yanqiu Sun, Jian Liu, Mingyu He, Xiaolu Chen

**Affiliations:** 1 Department of Rheumatism Immunity, The First Affiliated Hospital, Anhui University of Chinese Medicine, Hefei, Anhui, China; 2 Anhui Provincial Key Laboratory for Applied Basic and Clinical Translational Research on Rheumatologic Diseases in Traditional Chinese Medicine, Hefei, Anhui, China

**Keywords:** baicalin, ceRNA, Circ_0000734, inflammatory response, rheumatoid arthritis

## Abstract

**Background:**

Rheumatoid arthritis (RA) is a chronic autoimmune and inflammatory disease, and circular RNAs (circRNAs) are widely involved in its progression. Baicalin, a naturally derived small molecule, has been shown to improve inflammatory responses in RA by regulating circRNAs; however, the underlying regulatory mechanism remains unclear.

**Objective:**

This study aimed to investigate how circ_0000734 participates in the inflammatory response of RA-FLS through the miR-197-5p/IKBKB axis and to clarify the intervention effect of baicalin.

**Methods:**

The expression level of circ_0000734 in peripheral blood mononuclear cells (PBMCs) from RA patients was detected by qRT-PCR. Correlation analysis was performed to evaluate the associations between circ_0000734 expression and clinical indicators, including rheumatoid factor (RF), anti-cyclic citrullinated peptide antibody (anti-CCP), C-reactive protein (CRP), erythrocyte sedimentation rate (ESR), and DAS28 scores. The downstream targets miR-197-5p and IKBKB were predicted using bioinformatics databases. Dual-luciferase reporter assays were performed to verify the targeting relationship between circ_0000734 and miR-197-5p, as well as the binding relationship between miR-197-5p and IKBKB. Subsequently, circ_0000734 and miR-197-5p were silenced or overexpressed in a TNF-α-stimulated RA fibroblast-like synoviocyte (RA-FLS) model to validate their regulatory relationship. Flow cytometry (FCM), Cell Counting Kit-8 (CCK-8), reverse transcription quantitative polymerase chain reaction (RT-qPCR), Western blot (WB), and enzyme-linked immunosorbent assay (ELISA) were used to determine the effects of circ_0000734 and miR-197-5p silencing/overexpression, as well as baicalin treatment, on RA-FLS cell viability, cell cycle progression, the NF-κB signaling pathway, and the expression of inflammatory factors, including IL-17, IL-23, IL-4, and IL-10.

**Results:**

circ_0000734 was highly expressed in PBMCs from RA patients and was positively correlated with disease activity indicators, including RF, CCP, CRP, ESR, and DAS28 scores. In the TNF-α-stimulated RA-FLS cell model, overexpression of circ_0000734 promoted RA-FLS cell proliferation, activated the NF-κB pathway, and disrupted the balance between pro-inflammatory and anti-inflammatory factors, whereas circ_0000734 silencing exerted the opposite effects. Dual-uciferase reporter assays confirmed the targeting relationship between circ_0000734 and miR-197-5p. Overexpression of miR-197-5p reversed the effects of circ_0000734 overexpression on RA-FLS cell viability, the downstream target gene IKBKB, the NF-κB pathway, and inflammatory factors. In addition, baicalin treatment downregulated circ_0000734 expression, thereby inhibiting the activation of the NF-κB pathway, improving the inflammatory cytokine profile, and reversing the adverse phenotype induced by circ_0000734 overexpression.

**Conclusion:**

Highly expressed circ_0000734 in RA promotes NF-κB pathway activation by inhibiting miR-197-5p expression, thereby enhancing RA-FLS cell viability and promoting inflammatory cytokine secretion. Baicalin may inhibit the high expression of circ_0000734 and activation of the NF-κB signaling pathway, ultimately alleviating the inflammatory response in RA-FLS.

## Graphical Abstract

**Figure:**
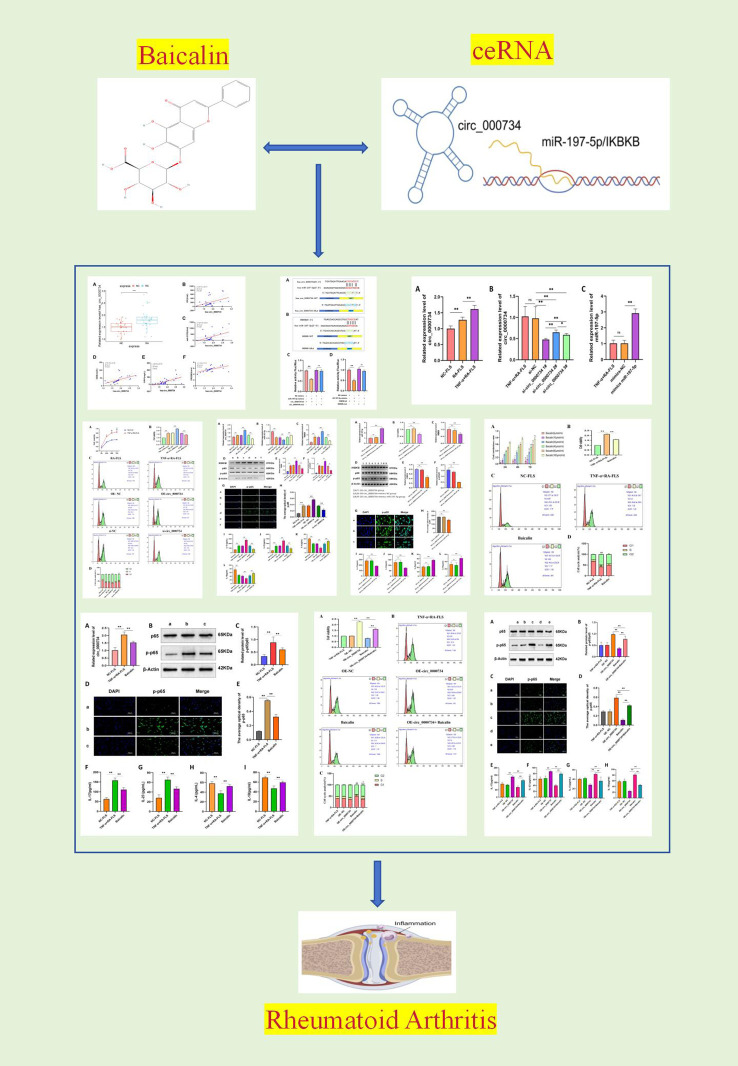

## 1 Introduction

Rheumatoid arthritis (RA) is a chronic, systemic autoimmune inflammatory disease characterized mainly by symmetrical joint pain and swelling (1, 2). It is chronic, progressive, and destructive, and may ultimately lead to joint deformity and functional disability. In recent years, the disease burden of RA has continued to increase. Epidemiological studies have shown that the global age-standardized prevalence of RA is 208.8 cases per 100,000 population, with women affected two to three times more frequently than men. The peak incidence occurs at 60–65 years of age in men and 55–60 years of age in women (3, 4). Predictions based on the Global Burden of Disease (GBD) database indicate that RA will remain a persistent public health challenge in the coming decade, with 317,000 people worldwide expected to be affected by RA by 2050 (5). These findings highlight the importance of early diagnosis and timely, effective treatment strategies for RA.

Although the pathogenesis of RA has not been fully elucidated, accumulating evidence suggests that genetic factors, environmental factors, immune dysregulation, and epigenetic modifications jointly contribute to its development (6, 7). Circular RNAs (circRNAs) are a special class of non-coding RNAs characterized by covalently closed loop structures (8). CircRNAs were first discovered in 1976 and were initially considered byproducts of splicing errors (9). With the development and widespread application of high-throughput sequencing and bioinformatics technologies, increasing evidence has demonstrated that circRNAs are widely present in eukaryotes (10, 11). Unlike linear RNAs generated by canonical splicing, circRNAs are produced through back-splicing and lack 5′ and 3′ termini, which makes them resistant to exonuclease-mediated degradation and confers high stability and conservation (8). MicroRNAs (miRNAs) are short, single-stranded non-coding RNAs of approximately 22 nucleotides that promote mRNA degradation or inhibit translation by binding to complementary sequences in the 3′ untranslated regions of target mRNAs (12–14). The competing endogenous RNA (ceRNA) hypothesis provides a novel framework for investigating intergenic regulatory mechanisms. Molecules such as circRNAs and mRNAs can act as miRNA “sponges” by competitively binding shared miRNAs through miRNA response elements, thereby indirectly reducing miRNA-mediated negative regulation of target mRNAs. This mechanism facilitates a deeper understanding of gene function and its regulatory role in disease progression (15, 16). Therefore, exploring the pathogenesis of RA from the perspective of circRNA-mediated ceRNA regulation and identifying potential therapeutic targets are of great significance. To identify circRNAs specifically expressed in RA, we previously performed high-throughput sequencing and bioinformatics analyses of circRNAs in peripheral blood mononuclear cells (PBMCs) from three RA patients and three healthy controls, and identified circ_0000734 as a differentially expressed circRNA in RA patients (17). However, the specific mechanism by which circ_0000734 participates in RA regulation as a ceRNA remains unclear.

There is currently no definitive curative treatment for RA. Existing therapeutic strategies, including disease-modifying antirheumatic drugs (DMARDs), such as methotrexate, hydroxychloroquine, and sulfasalazine, as well as nonsteroidal anti-inflammatory drugs (NSAIDs) and glucocorticoids (GCs), are associated with frequent adverse reactions and drug resistance (18, 19). Although biologic and targeted synthetic DMARDs have emerged as promising therapeutic options, their clinical application remains limited by high costs and accessibility issues for many patients (20). Therefore, identifying novel therapeutic targets and effective drugs remains an urgent need in the clinical management of RA. In recent years, with the continued development of traditional Chinese medicine research, the therapeutic role of bioactive components in RA has been widely recognized. Moreover, these bioactive components have shown great potential in the treatment of RA by regulating circRNA expression (21).

Baicalin is a flavonoid compound isolated from *Scutellaria baicalensis* and has anti-inflammatory, antioxidant, and anti-apoptotic effects. It plays an important role in the treatment of RA, systemic lupus erythematosus (SLE), and other rheumatic and immune-related diseases (22, 23). A study using pristane-induced lupus mice showed that baicalin reduced the production of pro-inflammatory cytokines, including TNF-α and IL-6, as well as PGE2, and inhibited abnormal T-cell activation (24). Another randomized, double-blind, placebo-controlled trial showed that baicalin significantly reduced blood lipid levels and inflammatory status in patients with RA (25). These findings provide evidence supporting the potential of baicalin to improve inflammatory responses in patients with RA. However, the specific mechanism by which baicalin regulates the inflammatory response in RA through circ_0000734 remains to be further clarified.

In this study, based on our previous findings, we further expanded the clinical sample size to examine the expression of circ_0000734 in patients with RA. Combined with bioinformatics analysis and dual-luciferase reporter assays, we identified miR-197-5p as a potential binding partner of circ_0000734 and IKBKB as a potential target gene of miR-197-5p. Subsequently, in a TNF-α-stimulated RA-FLS cell model, we explored the regulatory role of circ_0000734 and its target miR-197-5p in RA-associated inflammatory responses through circ_0000734 and miR-197-5p knockdown or overexpression, thereby clarifying the intervention mechanism of baicalin. The specific research design is shown in **Graphical Abstract**.

**Figure 1:**
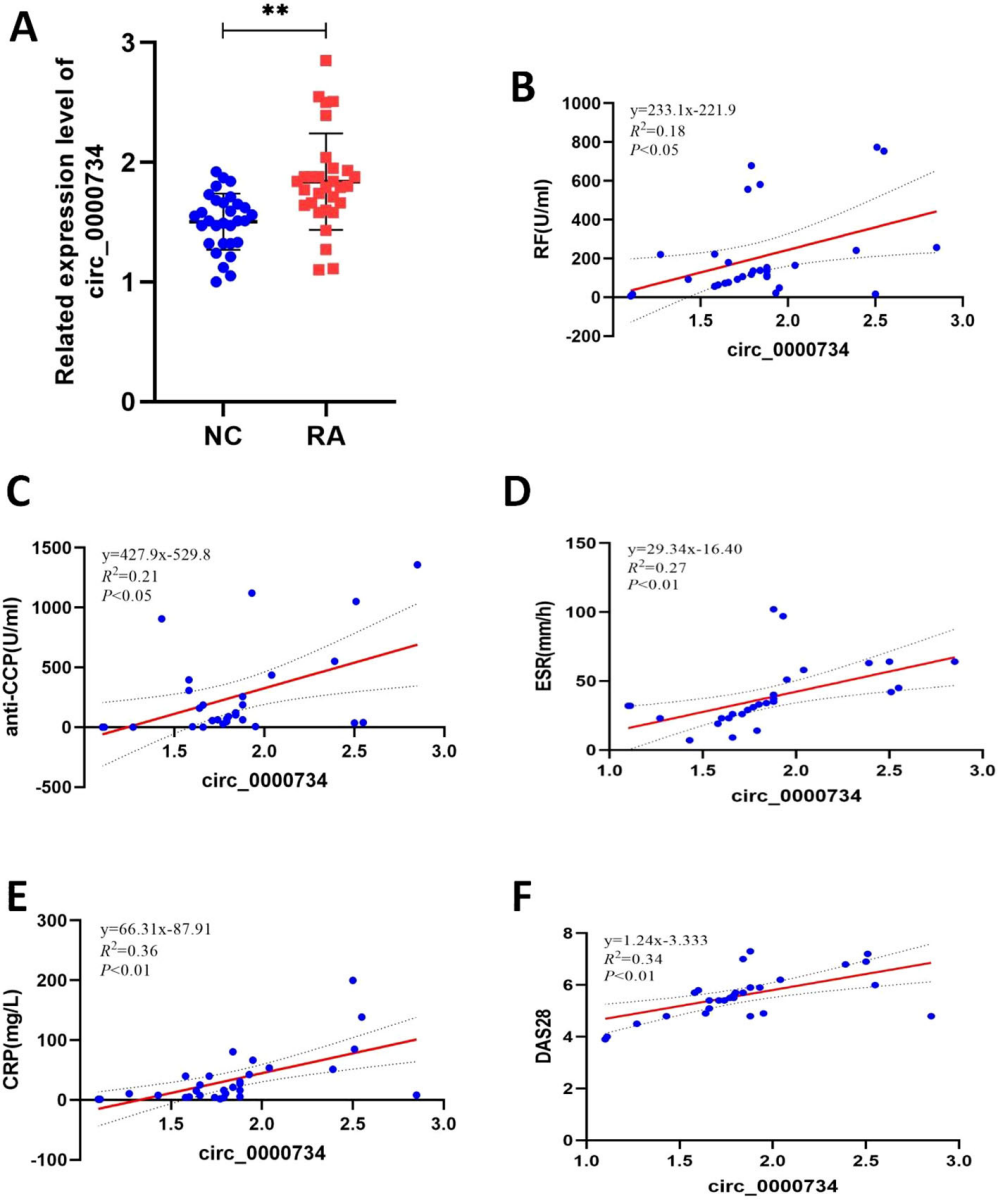
circ_0000734 expression and its correlation with laboratory indicators in patients with RA. **(A)** **P < 0.01 labor. **(B–F)** Correlation analysis between circ_0000734 and laboratory indicators in RA patients.

## 2 Materials and methods

### 2.1 Clinical information of RA patients

A total of 30 patients with RA were recruited from the Department of Rheumatology, the First Affiliated Hospital of Anhui University of Chinese Medicine, including 4 males and 26 females. The mean age was 56.72 ± 11.87 years, and the mean disease duration was 3.52 ± 1.69 years. All patients met the 2010 RA classification criteria proposed by the American College of Rheumatology and the European League Against Rheumatism (26). Peripheral blood samples were collected from the patients, and laboratory indicators, including rheumatoid factor (RF), anti-cyclic citrullinated peptide antibody (anti-CCP), erythrocyte sedimentation rate (ESR), and C-reactive protein (CRP), were recorded. The Disease Activity Score in 28 joints (DAS28) was also calculated. Meanwhile, blood samples were collected from 30 healthy individuals for PBMC extraction. During information collection, patient privacy was protected, and the study did not interfere with the treatment plans. The requirement for informed consent was waived by the Ethics Committee of the First Affiliated Hospital of Anhui University of Chinese Medicine.

### 2.2 Correlation analysis and *post hoc* power analysis

Pearson correlation analysis was used to evaluate the strength and direction of the linear relationships between circ_0000734 expression and clinical indicators in RA patients, including RF, anti-CCP, CRP, ESR, and DAS28. The Pearson correlation coefficient ranges from -1.0 to 1.0, where -1.0 indicates a perfect negative correlation, 1.0 indicates a perfect positive correlation, and 0 indicates no linear correlation between two variables. Based on the correlation results, key variables, including RF, anti-CCP, CRP, ESR, and DAS28, were selected, and linear regression models were constructed to evaluate the influence of circ_0000734 expression on these variables. Finally, based on the observed effect sizes, the statistical power of each test was calculated using the “Correlation: Bivariate normal model” module in G*Power software. This analysis was performed to assess the robustness of the results and to identify findings whose interpretation might be limited by insufficient statistical power.

### 2.3 Drugs and reagents

Cell Counting Kit-8 (CCK-8; Cat. No. BA00208) was purchased from BIOSS (Beijing, China). RA-FLSs (HUM-iCell-S010RA, P2) and FLSs (HUM-iCell-S010, P2) were obtained from Saicom Bio-Technology Co., Ltd. (Shanghai, China). Fibroblast culture medium (Cat. No. PriMed-iCell-003) was obtained from iCell (Shanghai, China). The Cell Cycle Kit (Cat. No. BL114A) was purchased from Biosharp (Beijing, China). Lipo8000™ transfection reagent (Cat. No. C0533) and the Dual-Luciferase Reporter Assay Kit (Cat. No. RG027) were purchased from Beyotime Biotechnology (Shanghai, China). IL-17 ELISA kit (Cat. No. JYM0082Hu), IL-23 ELISA kit (Cat. No. JYM0077Hu), IL-4 ELISA kit (Cat. No. JYM0142Hu), and IL-10 ELISA kit (Cat. No. JYM0155Hu) were obtained from Wuhan Gene Beauty Technology Co., Ltd. (Wuhan, China). β-actin (Cat. No. TA-09), goat anti-mouse IgG (Cat. No. ZB-2305), and goat anti-rabbit IgG (Cat. No. B-2301) were obtained from ZSGB-BIO (Beijing, China). p-p65 (Cat. No. 3033S) and p65 (Cat. No. 8242T) were obtained from CST (Shanghai, China). IKBKB (Cat. No. ET1611-23) was purchased from HUABIO (Hangzhou, China). Triton X-100 (Cat. No. B025), goat serum blocking solution (Cat. No. B010), and antifade mounting medium (Cat. No. B024) were obtained from Ebiogo (Suzhou, China).

### 2.4 Cell model construction

The detailed procedure for constructing the cell model was described in our previously published study (27). Briefly, FLS cells were thawed, cultured, and passaged in fibroblast culture medium, and RA-FLS cells at passages 3–6 were selected for subsequent experiments. RA-FLS cells were stimulated with TNF-α (20 ng/mL) and incubated overnight at 37 C in a 5% CO_2_ incubator. Cells from each group were then collected for subsequent experiments.

### 2.5 Plasmid construction and cell transfection

The full-length sequence of circ_0000734 was obtained by PCR amplification and subsequently cloned into the eukaryotic expression vector pcDNA3.1 using double-enzyme digestion. After transformation, colony screening, and DNA sequencing verification, high-purity plasmids were extracted for subsequent experiments. For circ_0000734 knockdown, three small interfering RNA sequences targeting circ_0000734 and their corresponding negative control sequences were designed, and their transfection efficiencies were examined in TNF-α-stimulated RA-FLS cells (**Table 1**). In addition, miR-197-5p mimics were transfected into TNF-α-stimulated RA-FLS cells, and the transfection efficiency was evaluated. For overexpression experiments, the constructed pcDNA3.1-circ_0000734 plasmid and its empty vector control were used. Briefly, 4 μL of the overexpression plasmid was added to 250 μL of serum-free medium and gently mixed. After the transfection reagent was thoroughly mixed, 5 μL was diluted with 250 μL of serum-free medium and incubated at room temperature for 5 minutes. The diluted overexpression plasmid was then mixed with the diluted transfection reagent to a total volume of 500 μL and incubated at room temperature for 20 minutes. Subsequently, 500 μL of the transfection mixture was added to each well, and the cells were gently shaken and cultured at 37 C for 24 hours to detect circ_0000734 expression. The culture medium was replaced 4-6 hours after transfection. After 24 hours of transfection, the cells were digested with trypsin, washed twice with PBS, collected by centrifugation, and stored at -80 C for further analysis.

**Table 1:** Primer information for circ_0000734.

Gene	Forward (5′→3′)	Reverse (5′→3′)
circ_0000734-1	GCCCUAUAAUGAAGGCCAUTT	AUGGCCUUCAUUAUAGGGCTT
circ_0000734-2	AUGAAGGCCAUCAUGCAGATT	UCUGCAUGAUGGCCUUCAUTT
circ_0000734-3	CCUAUAAUGAAGGCCAUCATT	UGAUGGCCUUCAUUAUAGGTT
circ_0000734-NC	UUCUCCGAACGUGUCACGUTT	ACGUGACACGUUCGGAGAATT

### 2.6 Reverse transcription quantitative polymerase chain reaction (RT-qPCR)

Total RNA was extracted from cells in each group using TRIzol reagent. Subsequently, equal amounts of RNA, usually 1 μg, were reverse-transcribed to obtain cDNA templates for qPCR analysis. qPCR was performed using a real-time fluorescence quantitative PCR system with the SYBR Green I method. Each 20 μL reaction mixture contained 10 μL of 2× SYBR Green Premix Pro Taq HS qPCR premix, 0.8 μL each of forward and reverse primers (10 μM), 2.0 μL of cDNA template, and RNase-free water to a final volume of 20 μL. The reaction conditions were as follows: pre-denaturation at 95 C for 30 seconds, followed by 40 amplification cycles at 95 C for 5 seconds and 60 C for 30 seconds. Melting curve analysis was then performed to confirm the specificity of the amplification products. To ensure the accuracy of the quantitative results, the amplification efficiency of all primer pairs was validated. Briefly, cDNA templates were serially diluted 10-fold and subjected to qPCR amplification. Standard curves were generated by plotting the logarithm of the template concentration against the corresponding cycle threshold (Ct) values. Primer amplification efficiency (E) was calculated using the formula E=10 ^ (-1/slope) -1. All primer pairs used in this study showed standard curve correlation coefficients (R^2^) greater than 0.99, with amplification efficiencies ranging from 90% to 110%, meeting the requirements for quantitative analysis. The relative expression levels of target genes were calculated using the 2^-ΔΔCt^ method. β-actin was used as the internal reference gene for data normalization, and gene expression levels in the experimental groups were expressed as fold changes relative to the control group, which was set to 1.0. All primer sequences are listed in **Table 2**.

**Table 2:** Primer sequences.

Gene	Forward (5′→3′)	Reverse (5′→3′)
Hu-β-actin	CCCTGGAGAAGAGCTACGAG	GGAAGGAAGGCTGGAAGAGT
circ_0000734	ATTGGCAATCCAGTGCCCTA	ACGAGAGGGTTCAACTGTG
Hu-U6	CTCGCTTCGGCAGCACA	AACGCTTCACGAATTTGCGT
Hu-IKBKB	CCTTCAAGAGCCCAAGAGGA	AGCTGTTGTTTCGGAGGAGA
Hu-miR-197-5p	CTATTCGGGTAGAGAGGGCAGT	AGTGCAGGGTCCGAGGTATT
Hu-miR-197-5p RT	GTCGTATCCAGTGCAGGGTCCGAGGTATTCGCACTGGATACGACCCTCCC	

### 2.7 Dual luciferase reporter assay

Firstly, the circBank database (https://www.circbank.cn/#/home) (28) was used to predict potential miRNAs that could bind to circ_0000734, among which miR-197-5p was identified as a candidate target. Subsequently, the TargetScanHuman 8.0 database (https://www.targetscan.org/vert_80/) was used to predict potential mRNA targets of miR-197-5p. Based on the bioinformatics prediction results, luciferase reporter vectors containing the wild-type or mutant miR-197-5p binding sequences of circ_0000734 were constructed and designated as circ_0000734-WT and circ_0000734-MUT, respectively. Similarly, luciferase reporter vectors containing the wild-type or mutant miR-197-5p binding sequences in the 3′ untranslated region of IKBKB were constructed and designated as IKBKB-WT and IKBKB-MUT, respectively. Using Lipofectamine 3000 transfection reagent, the recombinant reporter plasmids were co-transfected with miR-197-5p mimics or mimic control into RA-FLSs. After 48 hours of culture, cells from each group were collected, and relative luciferase activity was detected and analyzed using a dual-luciferase reporter assay.

### 2.8 CCK-8 assay for cell viability

RA-FLS suspensions were prepared and seeded into plates, followed by incubation overnight at 37 C. After incubation for 12, 24, 48, and 72 hours, 10 μL of CCK-8 solution was added to each well, and the cells were further cultured for 4 hours. The absorbance of each well was measured at 450 nm using a microplate reader.

### 2.9 Flow cytometry analysis of the cell cycle

RA-FLS cells were collected by centrifugation and washed with PBS. A total of 1-10 × 10^5^ cells, including cells in the culture supernatant, were collected. The 5× binding buffer was diluted with double-distilled water to prepare a 1× working solution, and 500 μL of the 1× working solution was used to resuspend the cells. Annexin V-FITC (5 μL) and PI (10 μL) were added to each tube, mixed gently, and incubated at room temperature in the dark for 5 minutes. The cell cycle distribution of RA-FLS cells was then analyzed by FCM.

### 2.10 Enzyme-linked immunosorbent assay

The levels of IL-17, IL-23, IL-4, and IL-10 in the supernatants of RA-FLS cells were detected using corresponding ELISA kits according to the manufacturer’s instructions.

### 2.11 Western blot

FLS cells were lysed with RIPA lysis buffer (Biosharp), and total proteins were extracted. The primary antibodies used in this study included rabbit anti-p-p65 (1:1000), rabbit anti-p65 (1:1000), and rabbit anti-IKBKB (1:2000). We used Image J software to analyze the grayscale values of various protein bands and normalized the data as “target protein grayscale value/internal reference protein (β -actin) grayscale value”. Finally, we plotted the normalized data into a bar graph and labeled the statistically significant differences. A *p* value < 0.05 was considered statistically significant.

### 2.12 Immunofluorescence

Cells from each group were collected, fixed with 4% paraformaldehyde for 20 minutes, and washed three times with PBS-T. Subsequently, 0.5% Triton X-100 was added dropwise, and the cells were covered and incubated at 37 C for 30 minutes. After Triton X-100 was removed, the cells were washed three times with PBS-T. Goat serum blocking solution was then added, and the cells were incubated at 37 C. After blocking, the goat serum blocking solution was removed, and a sufficient amount of primary antibody against p-p65 was added. The cells were covered and incubated at 37 C for 60 minutes. After removal of the primary antibody, the cells were washed three times with PBS-T. A sufficient amount of IF secondary antibody, goat anti-rabbit IgG diluted at 1:400, was then added, and the cells were incubated at 37 C in the dark for 30 minutes. After removal of the secondary antibody, the cells were washed three times with PBS-T. The nuclei were stained with DAPI for 5 minutes in the dark. After washing with PBS, the samples were mounted, and images were captured under a fluorescence microscope.

### 2.13 Statistical methods

Data were analyzed using GraphPad Prism (version 9.0.0). Comparisons between two groups were performed using unpaired t-tests. Comparisons among multiple groups were conducted using one-way analysis of variance (ANOVA) or repeated-measures ANOVA, followed by Bonferroni correction for multiple comparisons. A *p* value < 0.05 was considered statistically significant.

## 3 Results

### 3.1 High circ_0000734 expression is associated with inflammatory responses and disease activity in RA patients

Analysis of PBMCs from 30 RA patients and 30 healthy controls showed that circ_0000734 was significantly upregulated in PBMCs from RA patients (**Figure 1A**). Correlation analysis showed that circ_0000734 expression was significantly correlated with RF, anti-CCP, ESR, CRP, and DAS28 scores (*p*<0.05). Among these indicators, circ_0000734 showed a strong correlation with CRP (r>0.6) and moderate correlations with RF, anti-CCP, ESR, and DAS28 scores (0.3<r<0.6). *Post hoc* power analysis showed that the statistical power for the observed effect sizes ranged from 0.767 to 0.979, indicating that this study had sufficient power to detect these effects. The detailed results are shown in **Supplementary Table 1**. In addition, linear regression analysis showed that increased circ_0000734 expression in RA patients was positively correlated with elevated RF, anti-CCP, ESR, CRP, and DAS28 scores (**Figures 1B–F**). The results showed that circ_0000734 was highly expressed in PBMCs of RA patients and positively correlated with RF, anti-CCP, CRP, ESR, and DAS28 scores.

### 3.2 Validation of the targeted interactions within the circ_0000734/miR-197-5p/IKBKB axis

**Figure 2:**
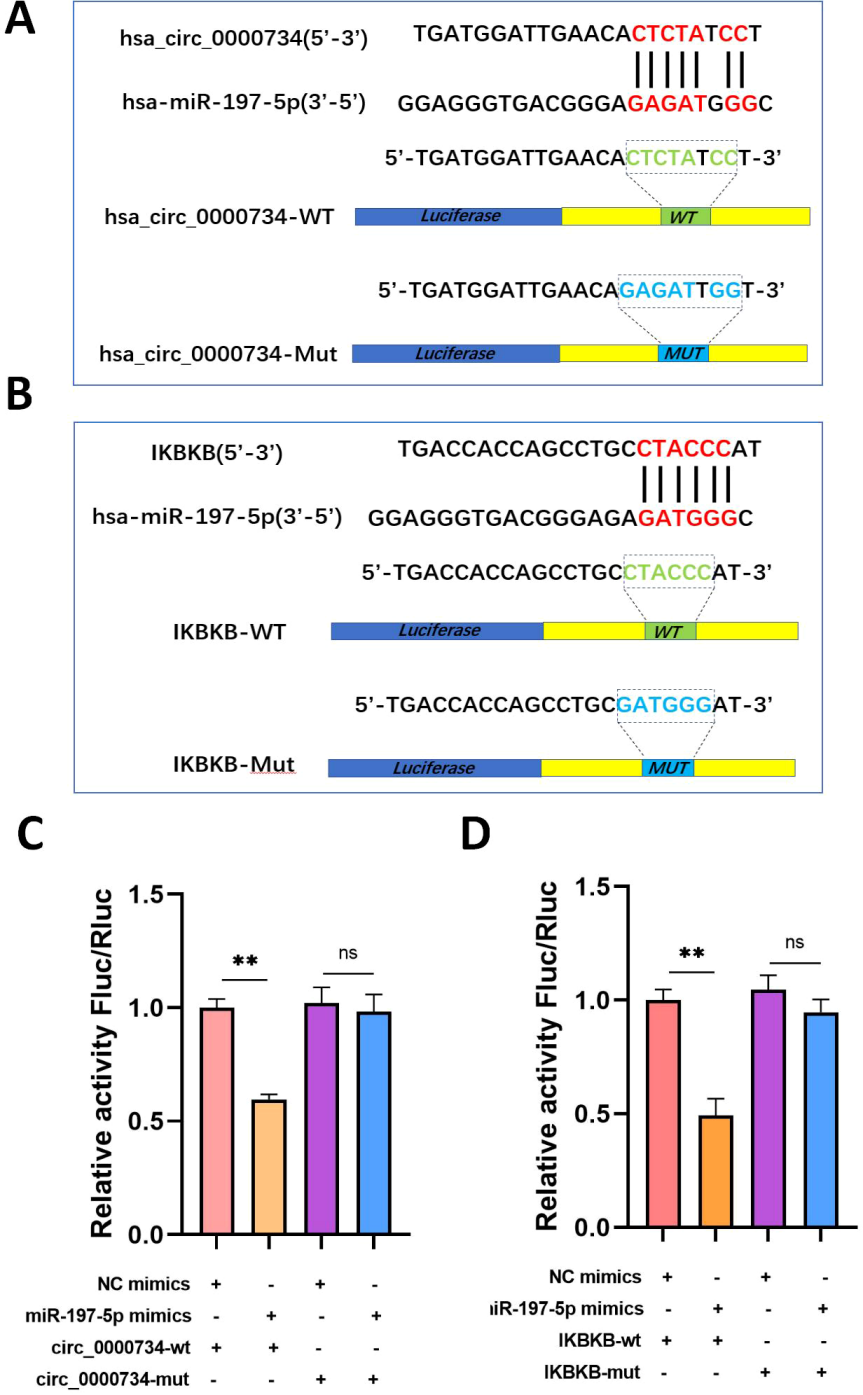
Targeted interactions within the circ_0000734/miR-197-5p/IKBKB axis. **(A)** Predicted binding sites between circ_0000734 and miR-197-5p. **(B)** Predicted binding sites between miR-197-5p and the target gene IKBKB. **(C)** Dual-luciferase reporter assay verifying the targeted interaction between circ_0000734 and miR-197-5p. **(D)** Dual-luciferase reporter assay verifying the targeted interaction between miR-197-5p and IKBKB. Intergroup comparisons were adjusted using Bonferroni correction for multiple comparisons. **P < 0.01, ns, not significant.

We first used the circBank database (https://www.circbank.cn/) (28) to predict miRNAs that may bind to circ_0000734. The results suggested that miR-197-5p may contain potential binding sites for circ_0000734, and the predicted binding region showed typical complementary pairing characteristics. These findings indicated that circ_0000734 may act as a ceRNA by sponging miR-197-5p. Subsequently, the TargetScanHuman 8.0 database (https://www.targetscan.org/vert_80/) was used to predict potential target genes of miR-197-5p. The analysis showed that the 3′ untranslated region (3′ UTR) of IKBKB, a key gene involved in inflammatory and cellular signaling pathways, contained a potential binding site complementary to miR-197-5p, as shown in **Figures 2A**, **B**. These bioinformatics predictions suggested that circ_0000734 may relieve the inhibitory effect of miR-197-5p on IKBKB by sponging miR-197-5p, thereby forming a potential ceRNA regulatory axis, namely the circ_0000734/miR-197-5p/IKBKB axis. To clarify the targeted interactions within this axis, dual-luciferase reporter assays were performed to verify the binding specificity of miR-197-5p to the predicted target sequences and its post-transcriptional regulation of downstream target genes signaling.

To confirm whether miR-197-5p specifically binds to the predicted site in circ_0000734, we constructed wild-type and mutant reporter vectors containing the predicted binding sequence, designated circ_0000734-WT and circ_0000734-MUT, respectively. As shown in **Figure 2C**, compared with NC mimics, co-transfection with miR-197-5p mimics significantly inhibited the luciferase activity of the circ_0000734-WT reporter plasmid (*P*<0.01), indicating that miR-197-5p directly binds to circ_0000734. However, when the predicted binding site in circ_0000734 was mutated, miR-197-5p had no significant effect on luciferase activity. These results confirmed that circ_0000734 directly interacts with miR-197-5p. Subsequently, to investigate the downstream functional target of miR-197-5p, we focused on IKBKB, a key kinase gene in the NF-κB pathway, and verified the predicted binding site within its 3′ UTR. The results showed that miR-197-5p overexpression also significantly inhibited the luciferase activity of the IKBKB-WT reporter plasmid (*P*<0.01), whereas this inhibitory effect disappeared after mutation of the binding site (*P* > 0.01). These findings confirmed that hsa-miR-197-5p directly targets both circ_0000734 and the 3′ UTR of IKBKB in a highly specific manner through its seed sequence and suppresses reporter gene expression. Together, these results indicate that miR-197-5p serves as a key molecular bridge linking circ_0000734 to the regulation of downstream NF-κB pathway activation. Therefore, we further explored the molecular mechanism by which the circ_0000734/miR-197-5p/IKBKB axis regulates the inflammatory response in RA through the NF-κB pathway.

### 3.3 Expression and transfection efficiency of circ_0000734 and miR-197-5p in the TNF-α-induced RA-FLS model

**Figure 3:**
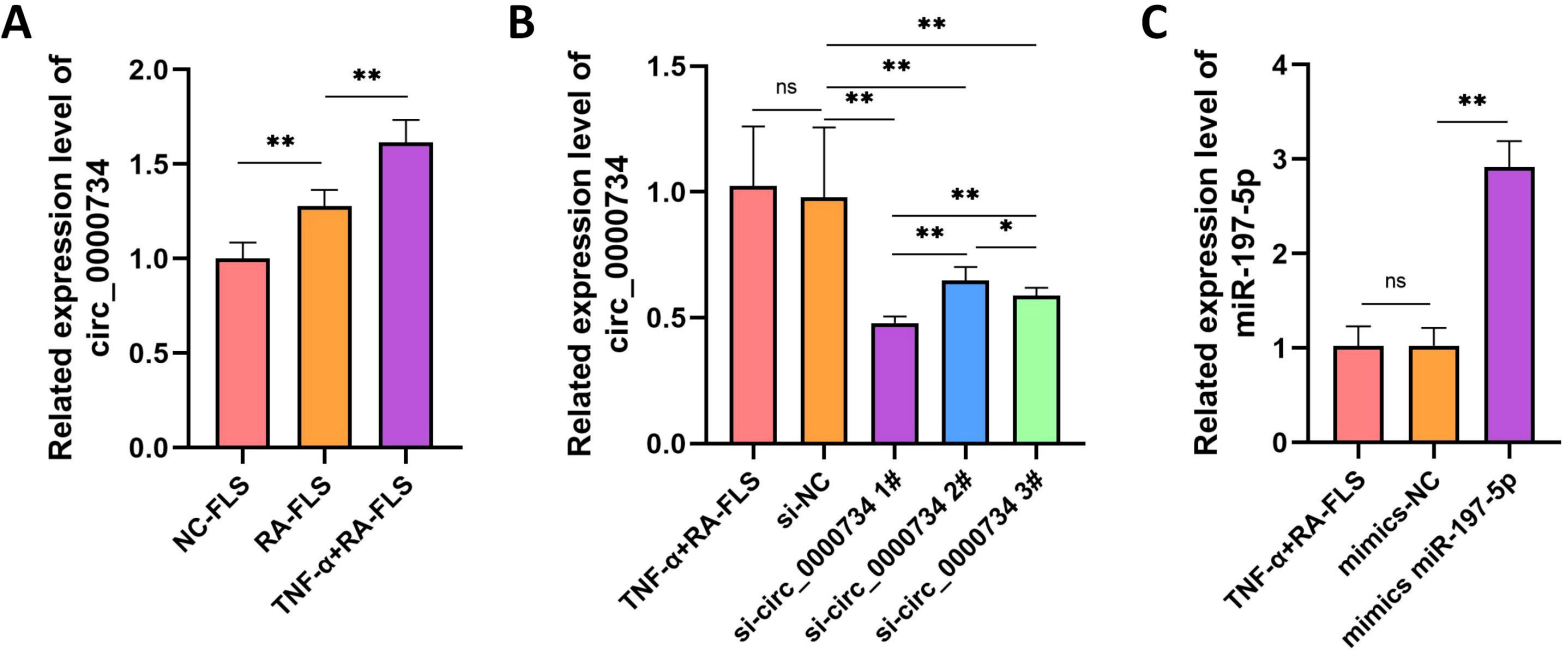
Expression and transfection efficiency of circ_0000734 and miR-197-5p in the TNF-α-induced RA-FLS model. **(A)** Changes in circ_0000734 expression in the disease model. **(B)** Validation of si-circ_0000734 transfection efficiency. **(C)** Validation of miR-197-5p mimic transfection efficiency. Intergroup comparisons were adjusted using Bonferroni correction for multiple comparisons. *P < 0.05, **P < 0.01, ns, not significant.

Firstly, the expression level of circ_0000734 was detected in the TNF-α-induced RA-FLS model. As shown in **Figure 3A**, circ_0000734 expression was significantly upregulated in RA-FLS cells compared with NC-FLS cells (*P <*0.01). Moreover, circ_0000734 expression was further increased in TNF-α-induced RA-FLS cells (*P <*0.01), indicating that circ_0000734 expression is positively regulated by the inflammatory microenvironment. Based on this model, RA-FLS cells were transfected with circ_0000734-specific siRNAs, including si-circ_0000734-1#, si-circ_0000734-2#, and si-circ_0000734-3#. The results showed that circ_0000734 expression was significantly reduced in all si-circ_0000734 groups compared with the si-NC group (*P <*0.01), confirming that circ_0000734 was effectively silenced. Among the three sequences, si-circ_0000734-1# showed the strongest knockdown efficiency; therefore, si-circ_0000734-1# was selected for subsequent experiments (**Figure 3B**). In addition, to verify the overexpression efficiency of miR-197-5p, miR-197-5p mimics were transfected into TNF-α-stimulated RA-FLS cells. RT qPCR analysis confirmed that the relative expression level of miR-197-5p was significantly increased after transfection with miR-197-5p mimics compared with the mimic NC group (*P <*0.01), indicating successful overexpression of miR-197-5p.

### 3.4 Effects of circ_0000734 knockdown or overexpression on RA-FLS cell viability and cell cycle progression

**Figure 4:**
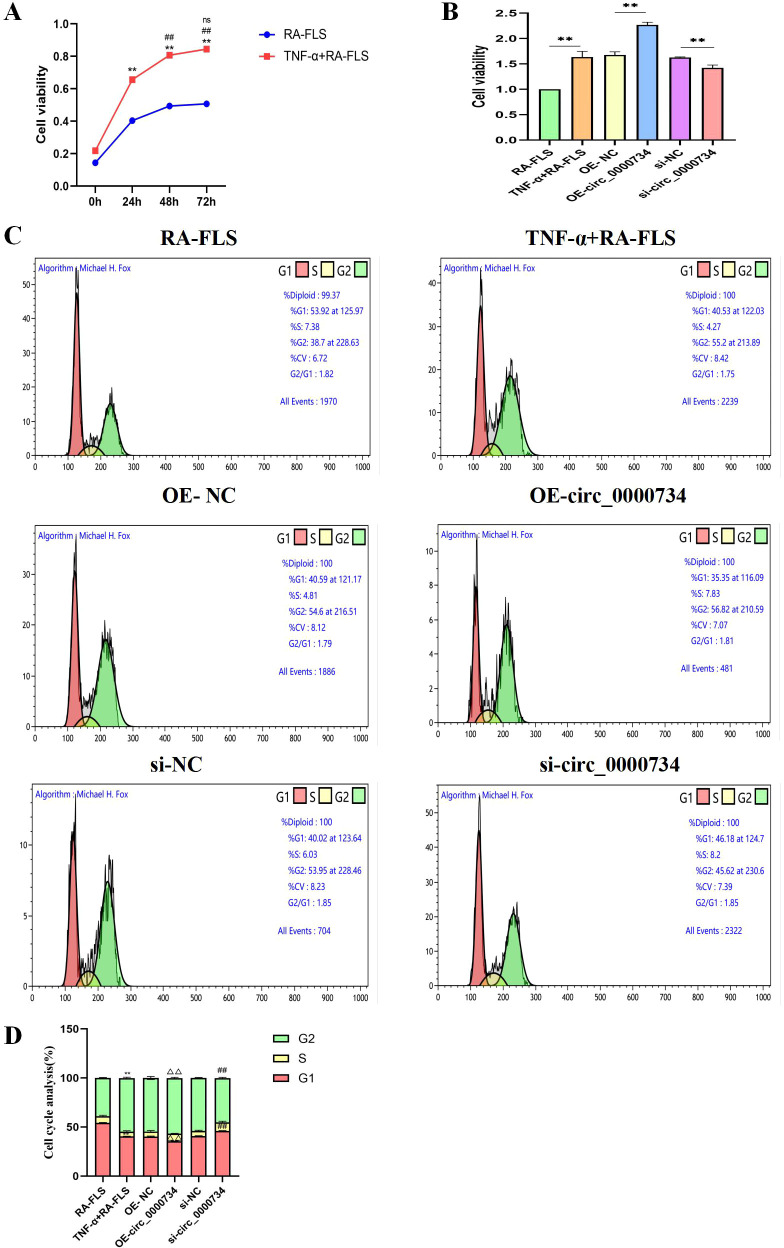
Effects of circ_0000734 knockdown or overexpression on RA-FLS cell function. **(A, B)** Cell viability was detected using the CCK-8 assay. **(C)** Cell cycle distribution was detected by FCM. **(A)** Compared to 0h ** P< 0.01, Compared to 24 hours ## P< 0.01, Compared to 48 hours, ns: no significance. **(B)** **P< 0.01. **(C)** **P < 0.01 vs. the RA-FLS. △△P < 0.01 vs. the pcNAD3.1-NC. ##P < 0.01 vs. the si-NC. **(C, D)** Cell cycle distribution was detected by FCM.

Cell viability and cell cycle progression are important indicators of cellular function and are essential for understanding cellular biological activity. In this study, CCK-8 and FCM assays were used to assess the viability and cell cycle distribution of RA-FLS cells. The CCK-8 assay showed that treatment with TNF-α at 20 ng/mL for 48 hours exerted the most significant effect on RA-FLS cell viability. Therefore, this concentration and treatment duration were selected for subsequent experiments (**Figure 4A**). The results showed that cell viability was significantly increased in the RA-FLS group and the pc-circ_0000734 group, whereas it was significantly decreased in the si-circ_0000734 group (**Figure 4B**). FCM analysis showed that the proportion of cells in the G1 phase was decreased, whereas the relative proportion of cells in the S+G2 phase was increased in the TNF-α+RA-FLS group, indicating that FLS cells were in a state of rapid proliferation. Compared with the pc-NC group, the pc-circ_0000734 group showed increased RA-FLS cell viability and a higher relative proportion of cells in the S+G2 phase (*P*<0.01). In contrast, compared with the si-NC group, the si-circ_0000734 group showed significantly reduced RA-FLS cell viability and a lower relative proportion of cells in the S+G2 phase. These results indicate that circ_0000734 overexpression promotes RA-FLS cell proliferation, whereas circ_0000734 knockdown inhibits RA-FLS cell proliferation (**Figures 4C, D**).

### 3.5 Effects of circ_0000734 knockdown or overexpression on miR-197-5p/IKBKB, p65 protein expression, and inflammatory cytokine production

**Figure 5:**
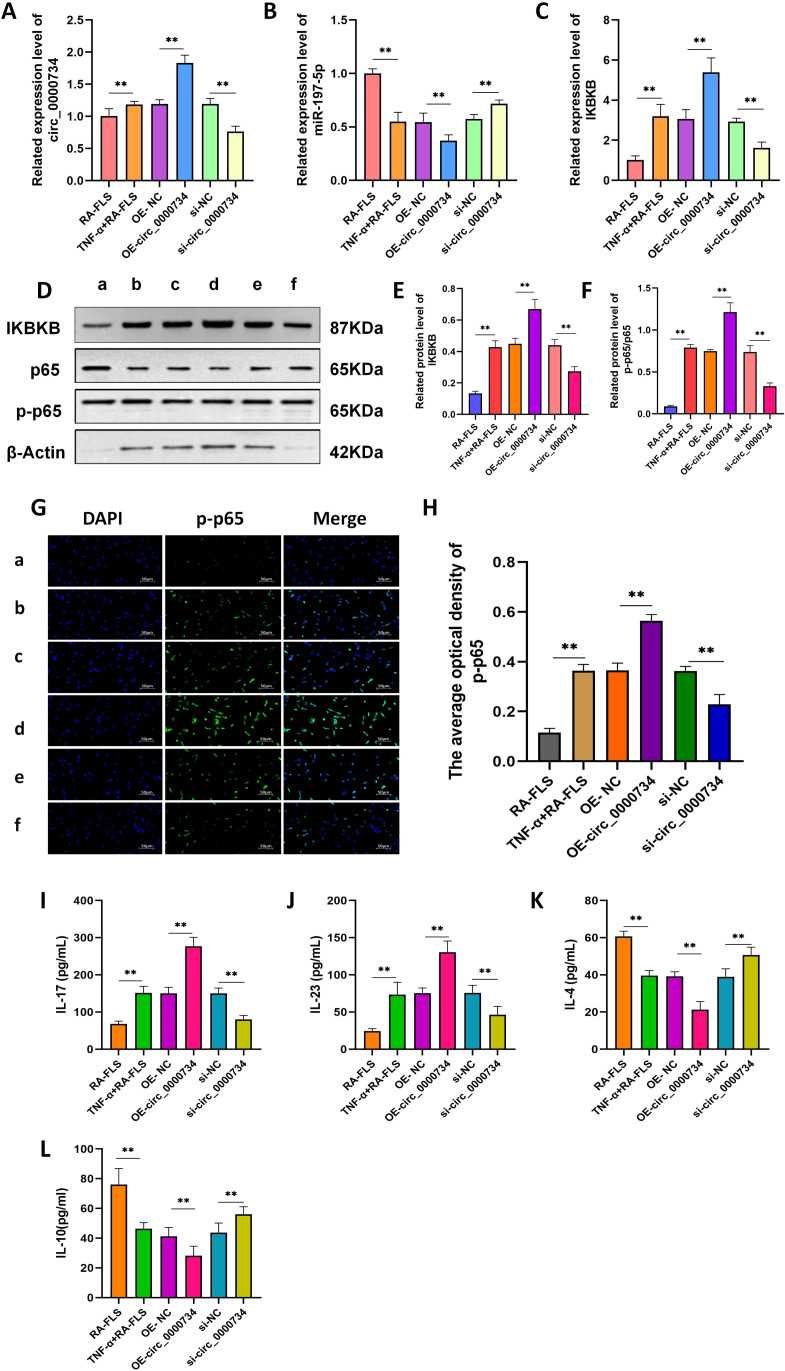
Effects of circ_0000734 knockdown or overexpression on miR-197-5p/IKBKB, p65 protein expression, and inflammatory cytokine production. **(A–C)** RT-qPCR was used to detect the expression levels of circ_0000734, miR-197-5p, and IKBKB. **(D)** WB analysis of IKBKB, p65, and p-p65 protein expression. **(E)** Relative protein expression level of IKBKB. **(F)** Changes in the p-p65/p65 protein ratio. **(G)** Immunofluorescence staining was used to observe p-p65 nuclear translocation. **(H)** Average optical density of p-p65 fluorescence staining. **(I–L)** ELISA was used to detect the expression levels of IL-17, IL-23, IL-4, and IL-10. a: RA-FLS; b: TNF-α+RA-FLS; c: OE-NC; d: OE-circ_0000734; e: si-NC; f: si-circ_0000734. Scale bar: 50 μm. Intergroup comparisons were adjusted using Bonferroni correction for multiple comparisons.**P < 0.01.

To further investigate the key mechanism by which circ_0000734 regulates the TNF-α-induced inflammatory response in RA-FLS cells, circ_0000734 was knocked down or overexpressed. We then examined the effects of circ_0000734 knockdown or overexpression on the expression levels of miR-197-5p and IKBKB mRNA, the protein levels of IKBKB, p65, and p-p65, as well as inflammatory cytokines, including IL-17, IL-23, IL-4, and IL-10, in RA-FLS cells. The results showed that circ_0000734 expression was significantly upregulated in RA-FLS cells under TNF-α stimulation. circ_0000734 overexpression inhibited endogenous miR-197-5p expression, while circ_0000734 knockdown produced the opposite effect, indicating a regulatory relationship between circ_0000734 and miR-197-5p (**Figures 5A–C**). Given that IKBKB is a direct target identified in our ceRNA network, we focus on its typical downstream effector p65 and changes in its phosphorylation level. The WB and IF staining confirmed that circ_0000734 relieved the inhibitory effect of miR-197-5p on IKBKB by downregulating miR-197-5p, leading to significantly increased IKBKB and p-p65 protein levels and promoting p65 nuclear translocation. These findings indicated activation of the NF-κB signaling pathway (**Figures 5D–I**). ELISA results further indicated that activation of the NF-κB signaling pathway increased the secretion of the pro-inflammatory cytokines IL-17 and IL-23, while inhibiting the production of the anti-inflammatory cytokines IL-4 and IL-10 (**Figures 5J–M**). These results suggest that highly expressed circ_0000734 may serve as a potential molecular target for RA treatment. Mechanistically, circ_0000734 may activate the NF-κB signaling pathway through the miR-197-5p/IKBKB network, disrupt the balance between pro-inflammatory and anti-inflammatory cytokines in RA synovial cells, and aggravate the inflammatory phenotype of RA-FLS cells.

### 3.6 Functional rescue verification of circ_0000734-mediated regulation of the RA-FLS inflammatory phenotype through miR-197-5p

**Figure 6:**
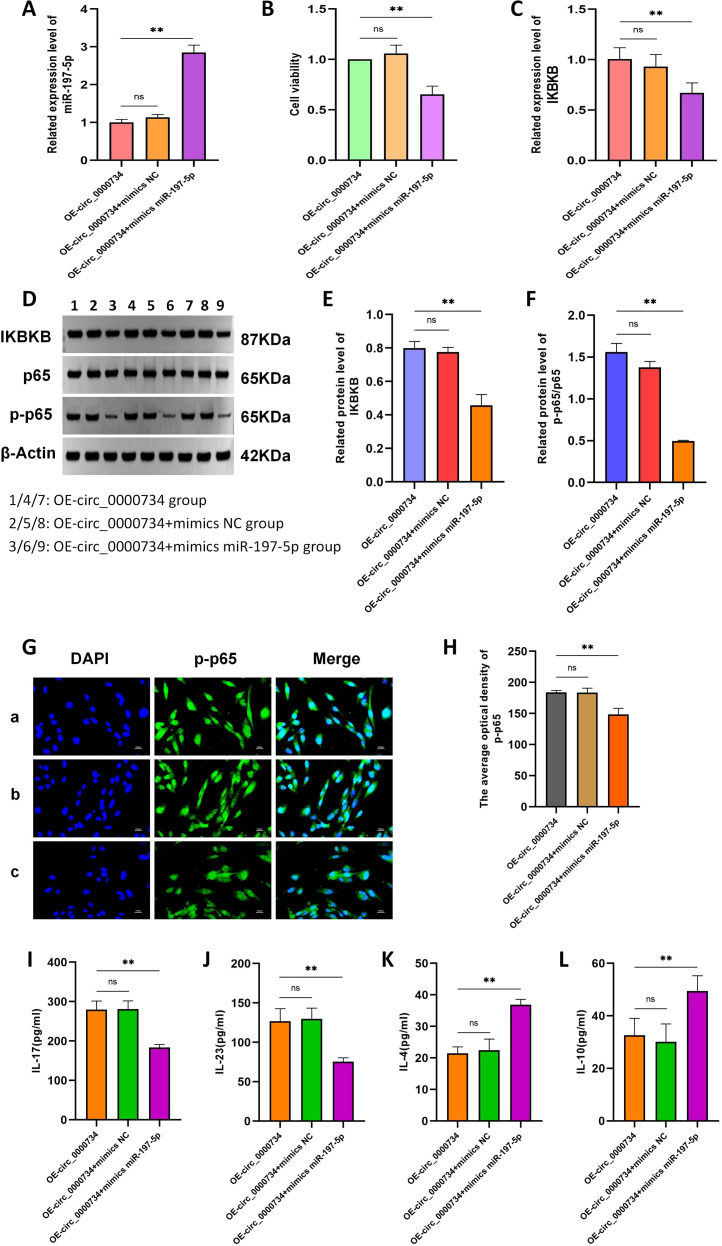
Functional rescue verification of circ_0000734-mediated regulation of the RA-FLS inflammatory phenotype through miR-197-5p. **(A)** RT-qPCR was used to detect miR-197-5p expression. **(B)** RA-FLS cell viability was detected using the CCK-8 assay. **(C)** RT-qPCR was used to detect IKBKB expression. **(D)** WB analysis of IKBKB, p65, and p-p65 protein expression. **(E)** Relative protein expression level of IKBKB. **(F)** Changes in the p-p65/p65 protein ratio. **(G)** Immunofluorescence staining was used to observe p-p65 nuclear translocation. **(H)** Average optical density of p-p65 fluorescence staining. **(I–L)** ELISA was used to detect the expression levels of IL-17, IL-23, IL-4, and IL-10. a: OE-circ_0000734; b: OE-circ_0000734 + mimic NC; c: OE-circ_0000734 + miR-197-5p mimic. Scale bar: 100 μm. Intergroup comparisons were adjusted using Bonferroni correction for multiple comparisons. **P < 0.01, ns, not significant.

To further investigate the molecular mechanism by which circ_0000734 regulates the IKBKB/NF-κB signaling pathway through the ceRNA mechanism, thereby affecting cellular function and inflammatory responses. miR-197-5p was overexpressed in circ_0000734-overexpressing RA-FLS cells (**Figure 6A**). The results showed that, compared with the OE-circ_0000734 group, there was no significant difference in cell viability in the OE-circ_0000734+mimic NC group. However, RA-FLS cell viability was significantly reduced in the OE-circ_0000734+mimic miR-197-5p group (*P <*0.01), indicating that overexpression of miR-197-5p reversed the promotive effect of circ_0000734 overexpression on RA-FLS cell viability (**Figure 6B**).

Subsequently, we further examined changes in IKBKB and p65 protein expression. The results showed that overexpression of miR-197-5p significantly reduced IKBKB expression in the context of circ_0000734 overexpression (**Figure 6C**). IF staining and WB results further confirmed that circ_0000734 overexpression may activate the NF-κB pathway by upregulating IKBKB and promoting p-p65, thereby inducing the release of the pro-inflammatory cytokines IL-17 and IL-23 and inhibiting the expression of the anti-inflammatory cytokines IL-4 and IL-10. However, miR-197-5p overexpression reversed these effects (**Figures 6D–L**). These results confirm that circ_0000734 promotes NF-κB pathway activation by suppressing miR-197-5p, thereby enhancing RA-FLS cell viability and inflammatory cytokine secretion. Exogenous overexpression of miR-197-5p reversed these effects, indicating that the circ_0000734/miR-197-5p axis plays an important role in regulating RA-FLS cell function and inflammatory responses.

### 3.7 Selection of the optimal baicalin concentration and its effects on RA-FLS cell viability and cell cycle progression

**Figure 7:**
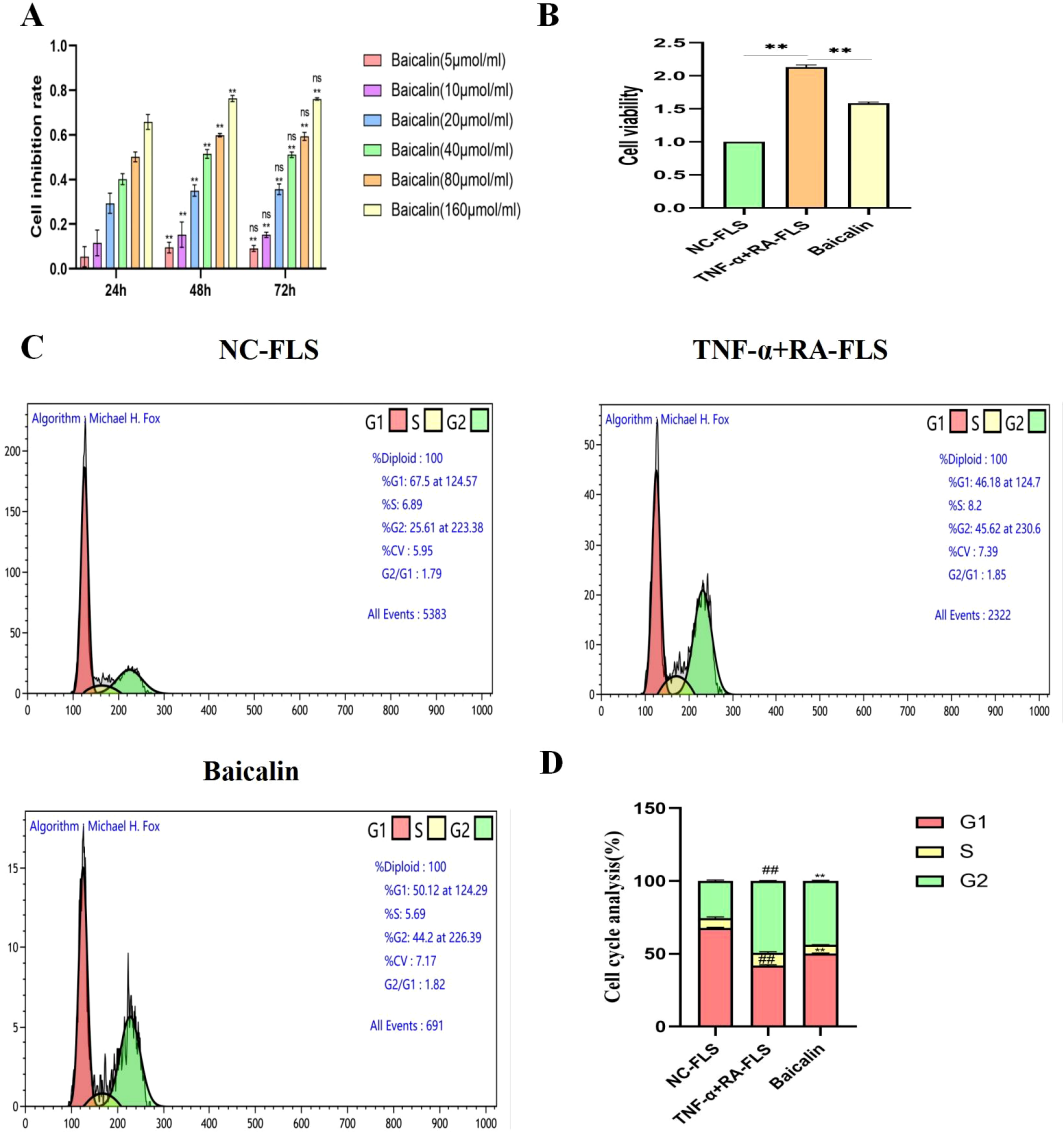
Optimal concentration of baicalin and its effects on RA-FLS cell function. **(A, B)** Cell viability was detected using the CCK-8 assay. **(C, D)** Cell cycle distribution was detected by FCM. Intergroup comparisons were adjusted using Bonferroni correction for multiple comparisons. **(A)** **P < 0.01 vs. 24h, ns vs. 48h, ns: no significance. **(B)** **P < 0.01. **(C)** ##P < 0.01 vs. the NC-FLS. **P < 0.01 vs. the TNF-α+RA-FLS.

The CCK-8 assay was used to evaluate the effects of different concentrations of baicalin (5, 10, 20, 40, 80, and 160 μmol/mL) on RA-FLS cell viability after treatment for 24, 48, and 72 hours, in order to determine the optimal concentration and treatment duration. The results showed that baicalin exerted the most significant inhibitory effect on RA-FLS cells at 48 hours. As the baicalin concentration increased, its inhibitory effect became more pronounced in a dose-dependent manner (*P*<0.01). The half-maximal inhibitory concentration (IC_50_) of baicalin was 45.507 μmol/mL. Therefore, 40 μmol/mL, which was close to the IC_50_ value, was selected as the optimal concentration of baicalin, and 48 hours was selected as the treatment duration for subsequent experiments (**Figure 7A**).

In addition, the CCK-8 results showed that cell viability was significantly increased in the TNF-α+RA-FLS group (*P*<0.01), whereas it was significantly decreased in the baicalin-treated group (*P*<0.01) (**Figure 7B**). FCM analysis showed that the relative proportion of cells in the S+G2 phase was significantly increased in the TNF-α+RA-FLS group (*P*<0.05), whereas it was significantly decreased after baicalin treatment (*P*<0.01) (**Figure 7C**). These results indicate that baicalin inhibits RA-FLS cell proliferation.

### 3.8 Effects of baicalin treatment on circ_0000734 expression, p65 protein activation, and inflammatory cytokine production

**Figure 8:**
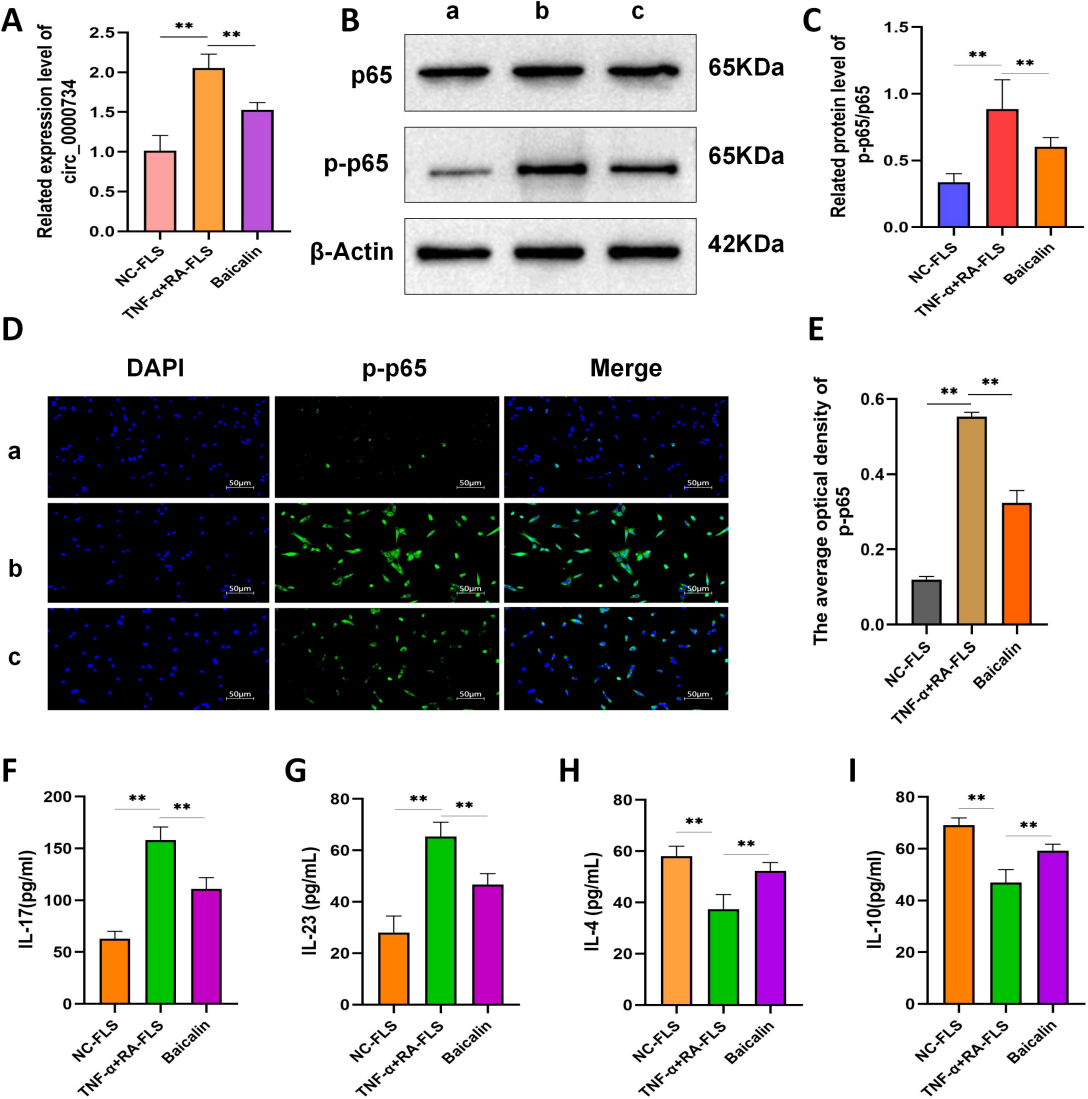
Effects of baicalin treatment on circ_0000734 expression, p65 protein activation, and inflammatory cytokine production. **(A)** RT-qPCR was used to detect circ_0000734 expression. **(B)** WB analysis of p65 and p-p65 protein expression. C: Changes in the p-p65/p65 protein ratio. **(D)** Immunofluorescence staining was used to observe p-p65 nuclear translocation. **(E)** Average optical density of p-p65 fluorescence staining. **(F–I)** ELISA was used to detect the expression levels of IL-17, IL-23, IL-4, and IL-10. a: RA-FLS; b: TNF-α+RA-FLS; c: baicalin. Scale bar: 50 μm. Intergroup comparisons were adjusted using Bonferroni correction for multiple comparisons. **P < 0.01.

RT-qPCR analysis showed that circ_0000734 expression was significantly increased in the TNF-α+RA-FLS group (*P*<0.01), whereas baicalin treatment significantly decreased circ_0000734 expression (*P*<0.01) (**Figure 8A**). IF staining and WB analysis showed that p65 translocated into the nucleus in the TNF-α+RA-FLS group, accompanied by increased p-p65/p65 protein expression. In contrast, after baicalin treatment, p65 nuclear translocation was reduced, and p-p65/p65 protein expression was downregulated (**Figures 8B, C**). These results indicate that baicalin inhibits circ_0000734 expression and suppresses activation of the NF-κB/p65 signaling pathway.

ELISA results showed that IL-17 and IL-23 expression levels were significantly increased in the TNF-α+RA-FLS group, whereas IL-4 and IL-10 expression levels were significantly decreased (*P*<0.01). After baicalin treatment, IL-17 and IL-23 expression levels were significantly reduced, while IL-4 and IL-10 expression levels were significantly increased (*P*<0.01) (**Figures 8D–G**). These results suggest that baicalin may inhibit the nuclear translocation of p65 protein and alleviate the inflammatory response in RA by downregulating the expression of circ_0000734.

### 3.9 Baicalin reverses the effects of circ_0000734 overexpression on RA-FLS cell viability and cell cycle progression

**Figure 9:**
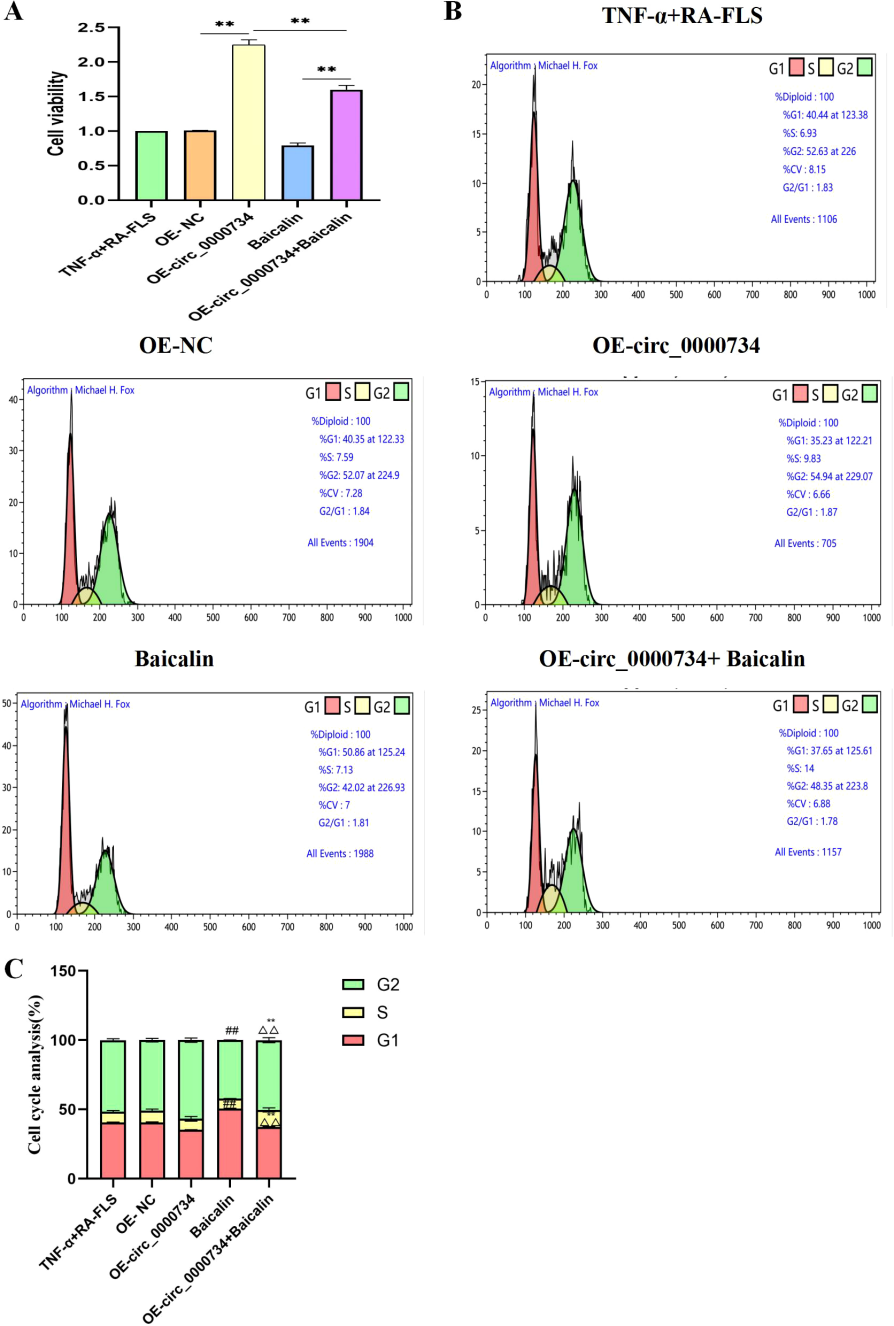
Baicalin reverses the effects of circ_0000734 overexpression on RA-FLS cell function. **(A)** Cell viability was detected using the CCK-8 assay. **P < 0.01. **(B, C)** Cell cycle distribution was detected by FCM. ##P < 0.01 vs. the OE-circ_0000734. **P < 0.01 vs. the Baicalin. △△P < 0.01 vs. the the OE-circ_0000734. ns, not significant.

The CCK-8 results showed that cell viability was significantly increased in the OE-circ_0000734 group (*P*<0.01), whereas baicalin treatment significantly reduced cell viability in OE-circ_0000734-transfected RA-FLS cells (*P*<0.01) (**Figure 9A**). FCM analysis showed that the relative proportion of cells in the S+G2 phase was significantly increased in the OE-circ_0000734 group (*P*<0.05), whereas it was significantly decreased after baicalin treatment (*P*<0.01) (**Figures 9B, C**). These results indicate that baicalin reverses the effects of circ_0000734 overexpression on RA-FLS cell viability and proliferation.

### 3.10 Baicalin reverses the effects of circ_0000734 overexpression on p65 nuclear translocation and inflammatory cytokine expression

**Figure 10:**
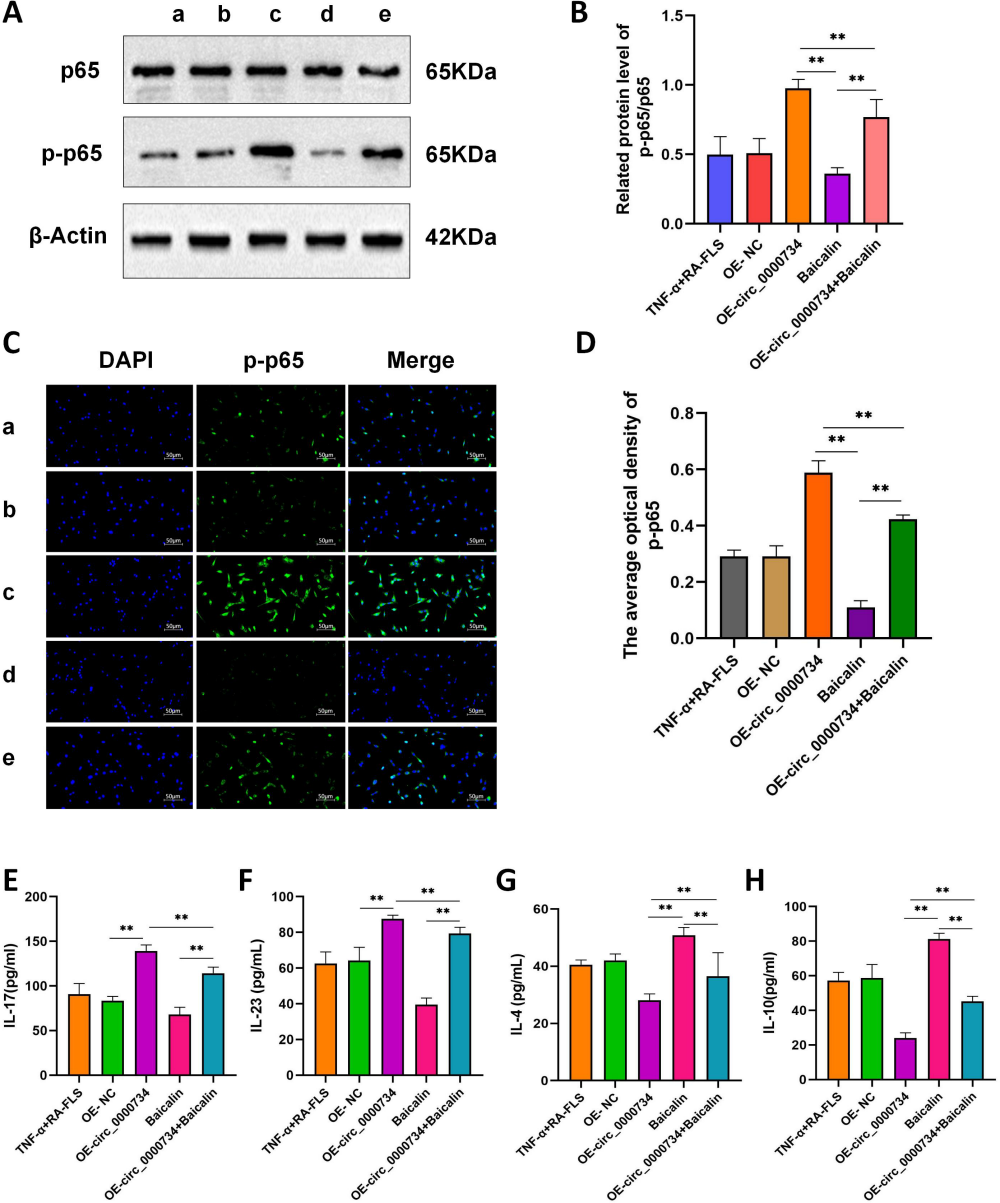
Baicalin reverses the effects of circ_0000734 overexpression on p65 nuclear translocation and inflammatory cytokine expression. **(A)** WB analysis of p65 and p-p65 protein expression. **(B)** Changes in the p-p65/p65 protein ratio. **(C)** Immunofluorescence staining was used to observe p-p65 nuclear translocation. **(D)** Average optical density of p-p65 fluorescence staining. **(E–H)** ELISA was used to detect the expression levels of IL-17, IL-23, IL-4, and IL-10. a, TNF-α+RA-FLS; b, OE-NC; c, OE-circ_0000734; d, baicalin; e, OE-circ_0000734 + baicalin. Scale bar: 50 μm. Intergroup comparisons were adjusted using Bonferroni correction for multiple comparisons. **P < 0.01.

IF staining and WB analysis showed that p65 translocated into the nucleus in the OE-circ_0000734 group, accompanied by increased p-p65/p65 protein expression. In contrast, after baicalin treatment in OE-circ_0000734-transfected RA-FLS cells, p65 nuclear translocation was reduced, and p-p65/p65 protein expression was decreased (**Figures 10A–D**). These results indicate that baicalin reverses circ_0000734 overexpression-induced activation of the NF-κB/p65 pathway.

ELISA results showed that IL-17 and IL-23 expression levels were significantly increased in the OE-circ_0000734 group, whereas IL-4 and IL-10 expression levels were significantly decreased (*P*<0.01). However, after baicalin treatment, IL-17 and IL-23 expression levels were significantly decreased, while IL-4 and IL-10 expression levels were significantly increased (*P*<0.01) (**Figures 10E–H**). These results suggest that baicalin reverses the effects of circ_0000734 overexpression on p65 nuclear translocation and inflammatory cytokine expression.

## 4 Discussion

In this study, analysis of PBMCs from clinical samples and cellular experiments demonstrated that circ_0000734 was highly expressed in RA patients. circ_0000734 activated the NF-κB signaling pathway by regulating miR-197-5p and IKBKB expression, thereby inducing inflammatory responses in RA. Baicalin reduced the expression of circ_0000734 and inhibited the activation of the NF-κB signaling pathway, thereby reducing the release of pro-inflammatory cytokines (IL-17, IL-23), while promoting the release of anti-inflammatory cytokines (IL-4, IL-10). These effects helped restore the dynamic balance of the inflammatory regulatory network in RA. This study is the first to link circ_0000734, NF-κB pathway activation, and the intervention effect of baicalin in an RA inflammatory model. Moreover, a complete ceRNA regulatory axis based on circ_0000734, namely the circ_0000734/miR-197-5p/IKBKB axis, was constructed, providing preliminary evidence for circ_0000734 as a potential therapeutic target for RA.

Inflammatory responses are central to the pathogenesis of RA and are closely associated with activation of the NF-κB/p65 pathway. NF-κB family member p65, also known as RelA, is a multifunctional transcription factor widely involved in various biological processes, including inflammation and immune regulation (29, 30). Activation of the p65 pathway is mainly mediated by the canonical IκB kinase (IKK) complex. Activation of the IKK complex leads to degradation of IκB proteins and subsequent release of p65, allowing p65 to translocate into the nucleus and regulate target gene expression (31, 32). In addition, p65 activation is also regulated by phosphorylation, with Ser276, Ser536, and Thr254 serving as key phosphorylation sites. The phosphorylation status of these sites significantly affects the stability, nuclear localization, and transcriptional activity of p65 (33, 34).

In RA, NF-κB/p65 can activate the expression of pro-inflammatory genes, participate in inflammatory signal transduction, and induce abnormal production of inflammatory cytokines. This process disrupts the dynamic balance between pro-inflammatory factors, including TNF-α, IL-17, and IL-23, and anti-inflammatory factors, including IL-10 and IL-4, thereby promoting the occurrence and progression of RA (35, 36). Imbalance in inflammatory cytokines can lead to abnormal immune responses in RA patients, followed by inflammatory cascade reactions that contribute to joint pain and tissue damage. Several inflammatory cytokines, including IL-6, IL-17, and TNF-α, have been identified as potential serum biomarkers for RA (37). IL-17 is a pro-inflammatory cytokine secreted by Th17 cells. It can activate Act1 by binding to its receptor, thereby inducing phosphorylation and degradation of IκBα, leading to NF-κB nuclear translocation and activation of inflammation-related gene expression. In contrast, IL-4, as an anti-inflammatory cytokine, can inhibit IL-17-induced NF-κB activation (38). IL-23 synergizes with IL-17 through the NF-κB signaling pathway, further aggravating the inflammatory response (39). IL-10 inhibits IκBα phosphorylation by binding to IL-10R, thereby preventing NF-κB translocation from the cytoplasm to the nucleus (40). In addition, IL-10 can directly inhibit IκBα degradation or indirectly suppress NF-κB activation by regulating other transcription factors, such as STAT3 (41, 42). Furthermore, pro-inflammatory factors such as IL-17 can induce the expression of receptor activator of nuclear factor-κB ligand (RANKL), which binds to RANK on the surface of osteoclast precursor cells, induces osteoclastogenesis, and promotes bone destruction and resorption (43, 44). Therefore, early control of immune-mediated inflammation is essential for preventing further RA progression, joint deformity, structural damage, and disability.

circRNAs are single-stranded, covalently closed RNA molecules generated from precursor mRNAs through a back-splicing mechanism. circRNAs can serve as miRNA sponges to regulate gene expression and protein interactions, thereby playing important roles in various biological processes (45). Previous studies have reported that other circRNAs, such as circRNA-0001283, are involved in various diseases, such as tumors and autoimmune diseases, by acting as miRNA sponges and regulating the NF-κB signaling pathway (45–48). These findings support the general view that circRNAs are key regulators of the NF-κB pathway. The innovation of this study lies in the first identification and confirmation of circ_0000734 as a novel functional regulator of the NF-κB p65 pathway in PBMCs and FLS cells from RA patients. Unlike previously reported circRNAs, circ_0000734 originates from the LOC100130691 locus, according to the circBase database, and may differ significantly in its sequence, expression profile in RA, and downstream miRNA/mRNA regulatory network. In addition, our study revealed for the first time that circ_0000734 is significantly overexpressed in PBMCs from RA patients, supporting its role as a key ceRNA involved in the regulation of RA inflammatory responses (31). In this study, clinical validation showed that circ_0000734 was significantly upregulated in PBMCs from RA patients, and its expression level was positively correlated with clinical disease activity indicators, including DAS28, and inflammatory markers, including RF, anti-CCP, CRP, and ESR. These findings suggest the potential of circ_0000734 as a biomarker of RA disease activity. Bioinformatics prediction further indicated that circ_0000734 may relieve the inhibitory effect of miR-197-5p on IKBKB by sponging miR-197-5p, thereby forming a potential ceRNA regulatory axis, namely circ_0000734/miR-197-5p/IKBKB axis. To further explore the mechanism by which this ceRNA regulatory axis modulates the RA inflammatory response in RA, we performed *in vitro* mechanistic experiments using TNF-α-stimulated RA-FLS cells. The results showed that overexpression of circ_0000734 enhanced RA-FLS cell viability by upregulating IKBKB and p-p65 protein expression, promoting p-p65 nuclear translocation, inducing the secretion of pro-inflammatory cytokines, including IL-17 and IL-23, and inhibiting the secretion of anti-inflammatory cytokines, including IL-4 and IL-10while. In contrast, overexpression of miR-197-5p reversed these effects. Together, these results demonstrate that the circ_0000734/miR-197-5p/IKBKB axis may participate in the regulation of RA-FLS cell function and inflammatory responses through the NF-κB pathway.

It is worth noting that this study not only identified a novel regulatory molecule but also further demonstrated that baicalin, an active component of traditional Chinese medicine, can target and downregulate circ_0000734, thereby inhibiting the NF-κB/p65 pathway and restoring the balance between pro-inflammatory and anti-inflammatory factors. Baicalin is a flavonoid compound extracted from the dried roots of *Scutellaria baicalensis* Georgi, a plant belonging to the Lamiaceae family. It exhibits a wide range of pharmacological activities, including anti-inflammatory, anti-apoptotic, and autophagy-regulating effects (49–51). Previous studies have reported that baicalin exerts anti-inflammatory effects by inhibiting the release of inflammatory cytokines, such as IL-6 and TNF-α, reducing the deposition of complement activation products, including C5a and C5b-9, and suppressing NF-κB p65 phosphorylation (52, 53). In this study, we further confirmed that baicalin inhibited the excessive proliferation of RA-FLS cells. After baicalin treatment, circ_0000734 expression in RA-FLS cells was decreased, accompanied by downregulation of p-p65/p65 protein expression and reduced levels of pro-inflammatory cytokines, including IL-17 and IL-23. Meanwhile, the expression of anti-inflammatory cytokines, including IL-4 and IL-10, was increased. These results indicate that baicalin can reverse pcDNA3.1-circ_0000734-induced activation of the NF-κB pathway and inhibit the inflammatory response in RA. This finding links a natural compound with clear clinical potential to a novel circRNA molecule within the RA inflammatory regulatory network, providing a new perspective for elucidating the molecular mechanism of baicalin in RA treatment. It also provides an experimental and theoretical basis for developing novel RA therapeutic strategies targeting circ_0000734.

It should be noted that the purpose of this study is to systematically confirm whether circ_0000734 can serve as a key and functional regulatory factor in the RA inflammatory network, and to clarify whether baicalin can inhibit the activation of the NF - κB pathway by targeting circ_0000734. Therefore, our current research mainly focuses on the validation of phenotype associations and ceRNA networks. To this end, we confirmed the functional causal relationship between high expression of circ_0000734 and activation of the NF - κB p65 pathway, as well as downstream inflammatory cytokine imbalance, through clinical association analysis of peripheral blood samples from RA patients and *in vitro* RA-FLS cell model studies. Baicalin intervention can reverse the above regulatory relationship. However, although this study provides strong theoretical evidence and a clear molecular hypothesis supporting circ_0000734 as a therapeutic target, these findings still need to be validated in *in vivo* animal models, such as collagen-induced arthritis (CIA) or adjuvant arthritis (AA) models. Further evaluation of the *in vivo* efficacy, pharmacokinetics, and safety of baicalin is needed to explore its potential clinical application. In addition, in this retrospective study, we evaluated the statistical power of the tests performed through *post hoc* power analysis, confirming heterogeneity in the ability of the fixed sample size of 30 cases to detect effects of different magnitudes. For the strong correlations identified, the relatively high statistical power (>0.95) increased the reliability of the correlation analysis results. However, for correlations of moderate intensity, the relatively low statistical power, such as that observed for RF (power = 0.767), suggests a potential risk of insufficient detection power in this study. Therefore, larger sample studies are needed in the future to more accurately estimate the true magnitude of these effects. Independent large-scale cohort studies are also required to verify the generalizability of the findings of this study.

## 5 Conclusion

This study demonstrates for the first time that circ_0000734 is highly expressed in RA and activates the NF-κB signaling pathway by sponging miR-197-5p and regulating IKBKB, thereby inducing an imbalance between pro-inflammatory and anti-inflammatory cytokines. Baicalin exerts anti-inflammatory effects by downregulating circ_0000734 and inhibiting NF-κB pathway activation. These findings provide evidence supporting circ_0000734 as a potential therapeutic target and biomarker for RA and offer a new perspective for elucidating the modern pharmacological mechanism of baicalin.

## Data Availability

The raw data supporting the conclusions of this article will be made available by the authors, without undue reservation.

## References

[1] Fraenkel L Bathon JM England BR St Clair EW Arayssi T Carandang K . 2021 American College of Rheumatology guideline for the treatment of rheumatoid arthritis. Arthrit Care Res. (2021) 73:924–39. doi: 10.1002/acr.24596. PMID: 34101387 PMC9273041

[2] Zhao J Guo S Schrodi SJ He D . Molecular and cellular heterogeneity in rheumatoid arthritis: mechanisms and clinical implications. Front Immunol. (2021) 12:790122. doi: 10.3389/fimmu.2021.790122. PMID: 34899757 PMC8660630

[3] Safiri S Kolahi AA Hoy D Smith E Bettampadi D Mansournia MA . Global, regional and national burden of rheumatoid arthritis 1990-2017: a systematic analysis of the Global Burden of Disease study 2017. Ann Rheum Dis. (2019) 78:1463–71. doi: 10.1136/annrheumdis-2019-215920. PMID: 31511227

[4] Zhou Y Luo X Li P Liu X Li J Su L . The burden of rheumatoid arthritis in China from 1990 to 2019 and projections to 2030. Public Health. (2023) 242:71–8. doi: 10.1016/j.puhe.2025.02.033. PMID: 40037154

[5] GBD 2021 Rheumatoid Arthritis Collaborators . Global, regional, and national burden of rheumatoid arthritis, 1990-2020, and projections to 2050: a systematic analysis of the Global Burden of Disease Study 2021. Lancet Rheumatol. (2023) 5:e594–610. doi: 10.1016/S2665-9913(23)00211-4. PMID: 37795020 PMC10546867

[6] D'Orazio A Cirillo AL Greco G Di Ruscio E Latorre M Pisani F . Pathogenesis of rheumatoid arthritis: one year in review 2024. Clin Exp Rheumatol. (2023) 42:1707–13. doi: 10.55563/clinexprheumatol/0307ed. PMID: 39315569

[7] McInnes IB Schett G . The pathogenesis of rheumatoid arthritis. New Engl J Med. (2011) 365:2205–19. doi: 10.1056/NEJMra1004965. PMID: 22150039

[8] Jeck WR Sorrentino JA Wang K Slevin MK Burd CE Liu J . Circular RNAs are abundant, conserved, and associated with ALU repeats. RNA. (2013) 19:141–57. doi: 10.1261/rna.035667.112. PMID: 23249747 PMC3543092

[9] Cocquerelle C Mascrez B Hétuin D Bailleul B . Mis-splicing yields circular RNA molecules. FASEB J. (1993) 7:155–60. doi: 10.1096/fasebj.7.1.7678559. PMID: 7678559

[10] Salzman J Gawad C Wang PL Lacayo N Brown PO . Circular RNAs are the predominant transcript isoform from hundreds of human genes in diverse cell types. PloS One. (2012) 7:e30733. doi: 10.1371/journal.pone.0030733. PMID: 22319583 PMC3270023

[11] Zhang Y Zhang XO Chen T Xiang JF Yin QF Xing YH . Circular intronic long noncoding RNAs. Mol Cell. (2013) 51:792–806. doi: 10.1016/j.molcel.2013.08.017. PMID: 24035497

[12] Tili E Michaille JJ Costinean S Croce CM . MicroRNAs, the immune system and rheumatic disease. Nat Clin Pract Rheumatol. (2008) 4:534–41. doi: 10.1038/ncprheum0885. PMID: 18728632

[13] Mucientes A Lisbona JM Mena-Vazquez N Ruiz-Limón P Manrique-Arija S Fernández-Nebro A . miRNA-mediated epigenetic regulation of treatment response in RA patients-a systematic review. Int J Mol Sci. (2022) 23:12989. doi: 10.3390/ijms232112989. PMID: 36361779 PMC9657910

[14] Wang J Yang H Ma H Han Q Li Q Ma J . miR-495 inhibits fibroblast-like synoviocyte proliferation and inflammation in rheumatoid arthritis rats via Wnt signaling pathway. Panminerva Med. (2022) 64:588–9. doi: 10.23736/s0031-0808.20.03855-0. PMID: 32077671

[15] Tay Y Rinn J Pandolfi PP . The multilayered complexity of ceRNA crosstalk and competition. Nature. (2014) 505:344–52. doi: 10.1038/nature12986. PMID: 24429633 PMC4113481

[16] Wang J Tan L Yu X Cao X Jia B Chen R . lncRNA ZNRD1-AS1 promotes Malignant lung cell proliferation, migration, and angiogenesis via the miR-942/TNS1 axis and is positively regulated by the m(6)A reader YTHDC2. Mol Cancer. (2022) 21:229. doi: 10.1186/s12943-022-01705-7. PMID: 36581942 PMC9801573

[17] Wen J Liu J Zhang P Jiang H Xin L Wan L . RNA-seq reveals the circular RNA and miRNA expression profile of peripheral blood mononuclear cells in patients with rheumatoid arthritis. Bioscience Rep. (2020) 40:1–11. doi: 10.1042/BSR20193160. PMID: 32191279 PMC7133114

[18] Xiong W Li Y Hu L He G Huang J . Risks of Malignancies related to disease-modifying antirheumatic drugs in rheumatoid arthritis: a pharmacovigilance analysis using the FAERS database. Front Pharmacol. (2023) 15:1458500. doi: 10.3389/fphar.2024.1458500. PMID: 39605908 PMC11598350

[19] Hai BB Anh TL Thi Thu PN Van HN Van GV Van DH . Latent and active tuberculosis development in patients with rheumatoid arthritis receiving biologic disease-modifying antirheumatic drugs: a single-center prospective study. PloS One. (2023) 19:e0295048. doi: 10.1371/journal.pone.0295048. PMID: 38206946 PMC10783715

[20] Russell MD Yang Z Dooley N Gibson M Zuckerman B Adas MA . Temporal and regional variation in the use of biologic and targeted synthetic DMARDs for rheumatoid arthritis: a nationwide cohort study. Rheumatology. (2023) 64(5):2432–41. doi: 10.1093/rheumatology/keae607. PMID: 39485485 PMC12048046

[21] Wang J Liu J Wen J Wang X . Triptolide inhibits inflammatory response and migration of fibroblast like synovial cells in rheumatoid arthritis through the circRNA 0003353/JAK2/STAT3 signaling pathway. Nan Fang Yi Ke Da Xue Xue Bao. (2022) 42:367–74. doi: 10.12122/j.issn.1673-4254.2022.03.08. PMID: 35426800 PMC9010992

[22] Yang J Yang X Yang J Li M . Baicalin ameliorates lupus autoimmunity by inhibiting differentiation of Tfh cells and inducing expansion of Tfr cells. Cell Death Dis. (2019) 10:140. doi: 10.1038/s41419-019-1315-9. PMID: 30760702 PMC6374440

[23] Chen X Wang Y Cai J Wang S Cheng Z Zhang Z . Anti-inflammatory effect of baicalin in rats with adjuvant arthritis and its autophagy- related mechanism. Technol Health Care. (2022) 30:191–200. doi: 10.3233/THC-228018. PMID: 35124596 PMC9028621

[24] Chae B . Effect of baicalin on the ex vivo production of cytokines in pristane-induced lupus mice. Yakhakhoe Chi. (2016) 60:21–8. doi: 10.17480/psk.2016.60.1.21

[25] Hang Y Qin X Ren T Cao J . Baicalin reduces blood lipids and inflammation in patients with coronary artery disease and rheumatoid arthritis: a randomized, double-blind, placebo-controlled trial. Lipids Health Dis. (2018) 17:146. doi: 10.1186/s12944-018-0797-2. PMID: 29935544 PMC6015450

[26] Aletaha D Neogi T Silman AJ Funovits J Felson DT Bingham CO . 2010 rheumatoid arthritis classification criteria: an American College of Rheumatology/European League Against Rheumatism collaborative initiative. Ann Rheum Dis. (2010) 69:1580–8. doi: 10.1136/ard.2010.138461. PMID: 20699241

[27] Sun Y Liu J Xin L Wen J Zhou Q Chen X . Xinfeng capsule inhibits inflammation and oxidative stress in rheumatoid arthritis by up-regulating LINC00638 and activating Nrf2/HO-1 pathway. J Ethnopharmacol. (2023) 301:115839. doi: 10.1016/j.jep.2022.115839. PMID: 36272490

[28] Liu M Wang Q Shen J Yang BB Ding X . Circbank: a comprehensive database for circRNA with standard nomenclature. RNA BIOL. (2019) 16(7):899–905. doi: 10.1080/15476286.2019.1600395 PMC6546381 31023147

[29] Yang QY Yang KP Li ZZ . MiR-22 restrains proliferation of rheumatoid arthritis by targeting IL6R and may be concerned with the suppression of NF-κB pathway. Kaohsiung J Med Sci. (2020) 36:20–6. doi: 10.1002/kjm2.12124. PMID: 31483954 PMC11896151

[30] Cao HY Li D Wang YP Lu HX Sun J Li HB . The protection of NF-κB inhibition on kidney injury of systemic lupus erythematosus mice may be correlated with lncRNA TUG1. Kaohsiung J Med Sci. (2020) 36:354–62. doi: 10.1002/kjm2.12183. PMID: 31930775 PMC11896366

[31] Aloor R Zhang C Bandyopadhyay M Dasgupta S . Impact of nuclear factor-κB on restoration of neuron growth and differentiation in hippocampus of degenerative brain. J Neurosci Res. (2015) 93:1471–5. doi: 10.1002/jnr.23547. PMID: 25586448

[32] Puar YR Shanmugam MK Fan L Arfuso F Sethi G Tergaonkar V . Evidence for the involvement of the master transcription factor NF-κB in cancer initiation and progression. Biomedicines. (2018) 6(3):1–21. doi: 10.3390/biomedicines6030082. PMID: 30060453 PMC6163404

[33] Kumar A Takada Y Boriek AM Aggarwal BB . Nuclear factor-kappaB: its role in health and disease. J Mol Med. (2004) 82:434–48. doi: 10.1007/s00109-004-0555-y. PMID: 15175863

[34] Kannan G Paul BM Thangaraj P . Stimulation, regulation, and inflammaging interventions of natural compounds on nuclear factor kappa B (NF-κB) pathway: a comprehensive review. Inflammopharmacology. (2023) 33:145–62. doi: 10.1007/s10787-024-01635-4. PMID: 39776026

[35] Noort AR Tak PP Tas SW . Non-canonical NF-κB signaling in rheumatoid arthritis: Dr Jekyll and Mr Hyde? Arthritis Res Ther. (2015) 17:15. doi: 10.1186/s13075-015-0527-3. PMID: 25774937 PMC4308835

[36] Maracle CX Kucharzewska P Helder B van der Horst C Correa de Sampaio P Noort AR . Targeting non-canonical nuclear factor-κB signalling attenuates neovascularization in a novel 3D model of rheumatoid arthritis synovial angiogenesis. Rheumatology. (2017) 56:294–302. doi: 10.1093/rheumatology/kew393. PMID: 27864565

[37] Grebenciucova E VanHaerents S . Interleukin 6: at the interface of human health and disease. Front Immunol. (2023) 14:1255533. doi: 10.3389/fimmu.2023.1255533. PMID: 37841263 PMC10569068

[38] Zhang P . Hypercoagulation in patients with rheumatoid arthritis correlates with activation of Act1/NF-κb signaling pathway. J Rheum Dis Treat. (2015) 1(4):1–10. doi: 10.23937/2469-5726/1510024

[39] Moschen AR Tilg H Raine T . IL-12, IL-23 and IL-17 in IBD: immunobiology and therapeutic targeting. Nat Rev Gastro Hepat. (2019) 16:185–96. doi: 10.1038/s41575-018-0084-8. PMID: 30478416

[40] Stanfield BA Purves T Palmer S Sullenger B Welty-Wolf K Haines K . IL-10 and class 1 histone deacetylases act synergistically and independently on the secretion of proinflammatory mediators in alveolar macrophages. PloS One. (2021) 16:e0245169. doi: 10.1371/journal.pone.0245169. PMID: 33471802 PMC7816993

[41] Rubtsov YP Rasmussen JP Chi EY Fontenot J Castelli L Ye X . Regulatory T cell-derived interleukin-10 limits inflammation at environmental interfaces. Immunity. (2008) 28:546–58. doi: 10.1016/j.immuni.2008.02.017. PMID: 18387831

[42] Huber S Gagliani N Esplugues E O'Connor W Huber FJ Chaudhry A . Th17 cells express interleukin-10 receptor and are controlled by Foxp3^−^ and Foxp3+ regulatory CD4+ T cells in an interleukin-10-dependent manner. Immunity. (2011) 34:554–65. doi: 10.1016/j.immuni.2011.01.020. PMID: 21511184 PMC3113617

[43] Wang K Li S Gao Y Feng X Liu W Luo R . BCL3 regulates RANKL-induced osteoclastogenesis by interacting with TRAF6 in bone marrow-derived macrophages. Bone. (2018) 114:257–67. doi: 10.1016/j.bone.2018.06.015. PMID: 29933112

[44] Rossini M Viapiana O Adami S Idolazzi L Fracassi E Gatti D . Focal bone involvement in inflammatory arthritis: the role of IL17. Rheumatol Int. (2016) 36:469–82. doi: 10.1007/s00296-015-3387-x. PMID: 26521079

[45] Zhang J Zhang Y Ma Y Luo L Chu M Zhang Z . Therapeutic potential of exosomal circRNA derived from synovial mesenchymal cells via targeting circEDIL3 /miR-485-3p /PIAS3 /STAT3 /VEGF functional module in rheumatoid arthritis. Int J Nanomedicine. (2021) 16:7977–94. doi: 10.2147/ijn.s333465. PMID: 34887661 PMC8651050

[46] Hu Y Guo F Zhu H Tan X Zhu X Liu X . Circular RNA-0001283 suppresses breast cancer proliferation and invasion via MiR-187/HIPK3 axis. Med Sci Monit. (2020) 26:e921502. doi: 10.12659/MSM.921502. PMID: 32066649 PMC7047918

[47] Yang J Lei X Zhang F . Identification of circRNA-disease associations via multi-model fusion and ensemble learning. J Cell Mol Med. (2024) 28:e18180–0. doi: 10.1111/jcmm.18180. PMID: 38506066 PMC10951890

[48] Li S Chen Q Liu Z Pan S Zhang S . Bi-SGTAR: a simple yet efficient model for circRNA-disease association prediction based on known association pair only. Knowledge-Based Syst. (2024) 291:111622. doi: 10.1016/j.knosys.2024.111622. PMID: 38826717

[49] Zhang Q Guo S Ge H Wang H . The protective role of baicalin regulation of autophagy in cancers. Cytotechnology. (2023) 77:33. doi: 10.1007/s10616-024-00689-0. PMID: 39760060 PMC11699138

[50] Zhang Q Guo S Wang H . The protective role of baicalin in the regulation of NLRP3 inflammasome in different diseases. Cell Biochem Biophys. (2023) 83(2):1–11. doi: 10.1007/s12013-024-01597-y. PMID: 39443419

[51] Yi Y Liu G Li Y Wang C Zhang B Lou H . Baicalin ameliorates depression-like behaviors via inhibiting neuroinflammation and apoptosis in mice. Int J Mol Sci. (2023) 25(19):10259. doi: 10.3390/ijms251910259. PMID: 39408591 PMC11476789

[52] Lu Y Zhou R Zhu R Wu X Liu J Ma Y . Baicalin ameliorates neuroinflammation by targeting TLR4/MD2 complex on microglia via PI3K/AKT/NF-κB signaling pathway. Neuropharmacology. (2023) 267:110296. doi: 10.1016/j.neuropharm.2025.110296. PMID: 39798687

[53] Zhang W Wang L Yang Y Ma Z Huang L Wan Q . Attenuation of the activation of NLRP3 inflammasome in fibroblast like synoviocytes of rheumatoid arthritis by baicalin through regulating the let-7i-3p/PI3K/Akt/NF-κB signaling axis. Medicinal Plant. (2024) 15(2):69–73. doi: 10.19601/J.CNKI.ISSN2152-3924.2024.02.018

