# Supplementary material for: Baicalin improves the inflammatory response of RA-FLS by targeting the circ_0000734/miR-197-5p/IKBKB axis
Source: Front Immunol. 2026 May 28;17:1828868. doi: 10.3389/fimmu.2026.1828868 (PMC13253440; doi:10.3389/fimmu.2026.1828868)
Supplement: Supplementary file 2 [file Table1.docx]

Supplementary Table 1. Post-event efficacy analysis

| **Gene** | **Index** | **Pearson correlation（r）** | **P** | **Statistical Effectiveness(1-β)** |
| --- | --- | --- | --- | --- |
| circ_0000734 | RF | 0.420 | 0.021 | 0.767 |
|  | ESR | 0.517 | 0.003 | 0.917 |
|  | CRP | 0.601 | 0.000 | 0.979 |
|  | CCP | 0.463 | 0.010 | 0.844 |
|  | DAS28 | 0.579 | 0.001 | 0.968 |

Note: N=30, α=0.05, two-tailed.
